# p37 regulates VCP/p97 shuttling and functions in the nucleus and cytosol

**DOI:** 10.1126/sciadv.adl6082

**Published:** 2024-05-03

**Authors:** Lidia Wrobel, Johanna L. Hoffmann, Xinyi Li, David C. Rubinsztein

**Affiliations:** ^1^ Department of Medical Genetics, Cambridge Institute for Medical Research, The Keith Peters Building, Cambridge Biomedical Campus, Hills Road, Cambridge CB2 0XY, UK.; ^2^ UK Dementia Research Institute, University of Cambridge, Cambridge Institute for Medical Research, The Keith Peters Building, Cambridge Biomedical Campus, Hills Road, Cambridge CB2 0XY, UK.

## Abstract

The AAA^+^-ATPase valosin-containing protein (VCP; also called p97 or Cdc48), a major protein unfolding machinery with a variety of essential functions, localizes to different subcellular compartments where it has different functions. However, the processes regulating the distribution of VCP between the cytosol and nucleus are not understood. Here, we identified p37 (also called UBXN2B) as a major factor regulating VCP nucleocytoplasmic shuttling. p37-dependent VCP localization was crucial for local cytosolic VCP functions, such as autophagy, and nuclear functions in DNA damage repair. Mutations in VCP causing multisystem proteinopathy enhanced its association with p37, leading to decreased nuclear localization of VCP, which enhanced susceptibility to DNA damage accumulation. Both VCP localization and DNA damage susceptibility in cells with such mutations were normalized by lowering p37 levels. Thus, we uncovered a mechanism by which VCP nucleocytoplasmic distribution is fine-tuned, providing a means for VCP to respond appropriately to local needs.

## Teaser

Nucleocytoplasmic shuttling and local functions of VCP/p97 are controlled by p37/UBXN2B.

## INTRODUCTION

Valosin-containing protein (VCP; also called p97, or Cdc48p in yeast) is an abundant, evolutionarily conserved AAA^+^ adenosine triphosphatase (ATPase) with two ATPase domains (D1 and D2) and a regulatory N-terminal domain, which forms homohexameric, ring-structured complexes. VCP serves as an integral chaperone for numerous quality control pathways, including endoplasmic reticulum (ER)–associated degradation (ERAD), macroautophagy, chromatin-associated protein degradation (CAD), and DNA damage repair (*1*, *2*). As a central component of the ubiquitin-proteasome system, VCP governs proteostasis by extracting and unfolding a variety of substrate proteins facilitating their subsequent degradation by the proteasome. In ERAD, VCP enables the translocation of misfolded proteins from the ER to the cytosol for their degradation. In the nucleus, VCP is crucial for a variety of protein degradation processes in which several chromatin-associated factors were identified as VCP substrates, including the DNA replicating licensing factor CDT1 (*3*, *4*) and various DNA double-strand break (DSB) repair proteins (*5*–*7*).

VCP also plays vital roles in distinct steps of macroautophagy (hereafter autophagy) (*8*–*10*). Recently, we identified VCP as a regulator of the early stages of autophagy initiation (*10*, *11*). Autophagosome formation is governed by the PI3K complex I consisting of Beclin-1, VPS15, VPS34, and ATG14L, as a defining subunit (*12*). VCP promotes the assembly and activity of the PI3K complex I thereby regulating the synthesis of phosphatidylinositol 3-phosphate [PI(3)P] on the autophagosome precursor membrane (*10*, *11*). Efficient PI(3)P production allows for the recruitment of downstream factors, such as WD repeat domain phosphoinositide-interacting protein 2 (WIPI2), and subsequent conjugation of LC3 family members to phosphatidylethanolamine in these membranes, a defining step in autophagosome formation.

The specific roles that VCP plays in cellular processes are controlled by a wide variety of cofactor proteins that directly bind to it (*13*, *14*). These cofactors are multidomain proteins composed of specific VCP-interacting modules and additional domains which, for example, function in the recognition of ubiquitylated target proteins (UBA domain). The majority of cofactors interact with the VCP N-terminal domain either via a UBX (ubiquitin regulatory X) domain or three linear binding motifs, called VCP-interacting motif, VCP-binding motif, and binding site 1 (BS1; also known as SHP box).

More than 45 missense mutations in VCP have been identified in multiple neurodegenerative diseases, including inclusion body myopathy associated with Paget disease of the bone and frontotemporal dementia (IBMPFD; also referred to as multisystem proteinopathy MSP1), amyotrophic lateral sclerosis (*15*), and tauopathy (*16*, *17*), indicating the importance of VCP function for neuronal health. Most of the disease-causing mutations are in the VCP N-D1 domain and result in conformational changes affecting its interaction with cofactors, thus perturbing downstream processes (*18*–*20*).

VCP resides in both the cytosol and the nucleus and different dominant mutations in VCP causing multisystem proteinopathy were reported to suppress the nuclear entry of VCP (*21*). However, the mechanism is not understood, and it is not known which factors regulate VCP shuttling between the nucleus and the cytosol in physiological conditions.

Here, we identified two VCP cofactors, p47 (also known as NSFL1C) and p37 (also known as UBXN2B), as major regulators of VCP nucleocytoplasmic shuttling. p37-mediated VCP shuttling affects its abilities to regulate autophagy and DNA damage repair pathways. In addition, we showed that VCP proteinopathy mutations cause the accumulation of DNA damage in human neurons which can be prevented by modulating the levels of p37.

## RESULTS

### p47 regulates the early stages of autophagosome biogenesis

**Fig. 1.**
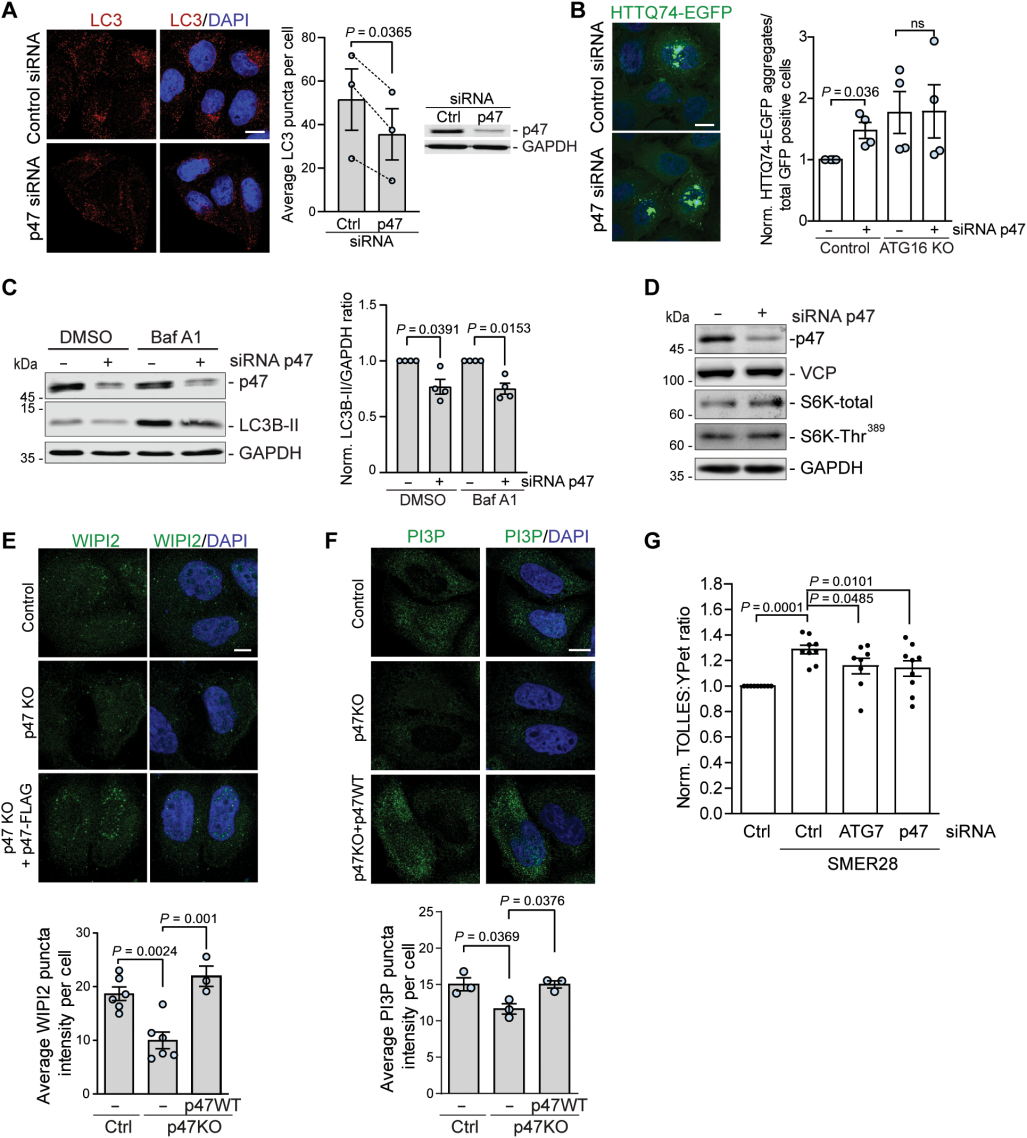
p47 regulates the early stages of autophagosome biogenesis. (**A**) LC3 puncta in control and p47 siRNA–treated HeLa cells, treated with 400 nM of BafA1 for 4 hours; *n* = 3, two-tailed paired Student’s *t* test. Data are means ± SEM. (**B**) HTTQ74-EGFP aggregates in control or ATG16 knockout (KO) HeLa cells, treated with p47 siRNA or control. The number of HTTQ74-EGFP aggregates represents the number of cells containing visible aggregates in cells positive for EGFP signal; *n* = 4, two-tailed paired Student’s *t* test. Data are means ± SEM. (**C**) LC3-II levels in control and p47 siRNA–treated HeLa cells, treated with 400 nM of BafA1 for 4 hours; *n* = 4, one-sample *t* test. Data are means ± SEM. (**D**) Protein levels in HeLa cells treated with control or p47 siRNA. (**E** and **F**) Control, p47 KO cells, or p47 KO cells expressing p47 wild-type (WT) were incubated in Earle’s balanced salt solution (EBSS) starvation media for 2 hours and stained for WIPI2 (E) or PI(3)P (F); *n* = 3 to 6, one-way ANOVA (*P* = 0.0005) with post hoc Tukey test for (E), one-way ANOVA (*P* = 0.0245) with post hoc Tuckey test for (F), data are means ± SEM. (**G**) HeLa cells expressing SRAI-LC3B were treated with control, p47, or ATG7 siRNA for 72 hours before treatment with 20 μM SMER28 for 24 hours, followed by fluorescence-activated cell sorting (FACS) analysis; *n* = 9, one-way ANOVA (*P* = 0.0005) with post hoc Tukey test, data are means ± SEM. Scale bars, 10 μm. In cDNA transfection experiments, matched empty vectors were used as controls for overexpression constructs, and in all knockdown experiments, we used nontargeting control siRNAs. ns, not significant; GAPDH, glyceraldehyde-3-phosphate dehydrogenase.

We recently reported an important role for VCP (also known as p97) in the early stages of autophagy initiation by showing that VCP ATPase activity is crucial for the formation of autophagic structures (*10*, *11*). Several studies have revealed that VCP ATPase activity is regulated by its cofactors p47 (NSFL1C) and p37 (UBXN2B) (*18*, *22*). Therefore, we tested if these cofactors affected autophagy. We observed a decrease in the numbers of autophagosomes, detected as LC3-positive puncta, in cells treated with small interfering RNA (siRNA) against p47 (Fig. 1A). Depletion of p47 increased the levels of a well-defined autophagy substrate, measured by the percentage of cells with mutant huntingtin exon 1 EGFP-HTT(Q74) [exon 1 of HTT with a 74-polyglutamine expansion fused at its N-terminal to enhanced green fluorescent protein (EGFP)] aggregates (Fig. 1B) (*23*). This was not observed in autophagy-deficient ATG16 knockout (KO) cells, further suggesting that decreased p47 levels compromise the autophagic process (Fig. 1B). To distinguish between effects on autophagosome formation and degradation, we depleted p47 using siRNA-mediated knockdown (Fig. 1C) or CRISPR-Cas9–mediated knockout (KO) (fig. S1, A and B) and analyzed cells treated with saturating concentrations of the lysosomal V-ATPase inhibitor bafilomycin A1 (BafA1), which blocks autophagosome degradation and allows one to infer autophagosome formation rates. In cells with and without BafA1, p47 depletion caused a decrease in the levels of the lipidated autophagosome membrane-bound form of LC3 (LC3-II) (Fig. 1C and fig. S1, A and B), which correlate with autophagosome volume/cell (*24*), indicating impaired autophagosome formation.

The impaired autophagosome biogenesis was not caused by decreased levels of VCP nor by changes in the activity of the mammalian target of rapamycin complex 1 as measured by the phosphorylation status of S6 kinase (S6K-Thr^389^; Fig. 1D). We found that depletion of p47 significantly impaired the early stage of autophagy initiation, including the formation of WIPI2 (Fig. 1E) and PI(3)P puncta (Fig. 1F), which could be rescued by exogenous expression of p47, confirming that the effect is specific. We have recently reported that activation of the VCP D1 ATPase by SMER28 induces autophagic flux specifically by stimulating PI(3)P production through PI3K complex I (*11*). Depletion of p47, in line with depletion of an important autophagy protein ATG7, diminished both steady-state (fig. S1, C and D) and SMER28-induced autophagic flux (Fig. 1G), suggesting that p47 is an important regulator VCP-dependent autophagosome biogenesis.

### p47 regulates autophagy through direct stabilization of p37

**Fig. 2.**
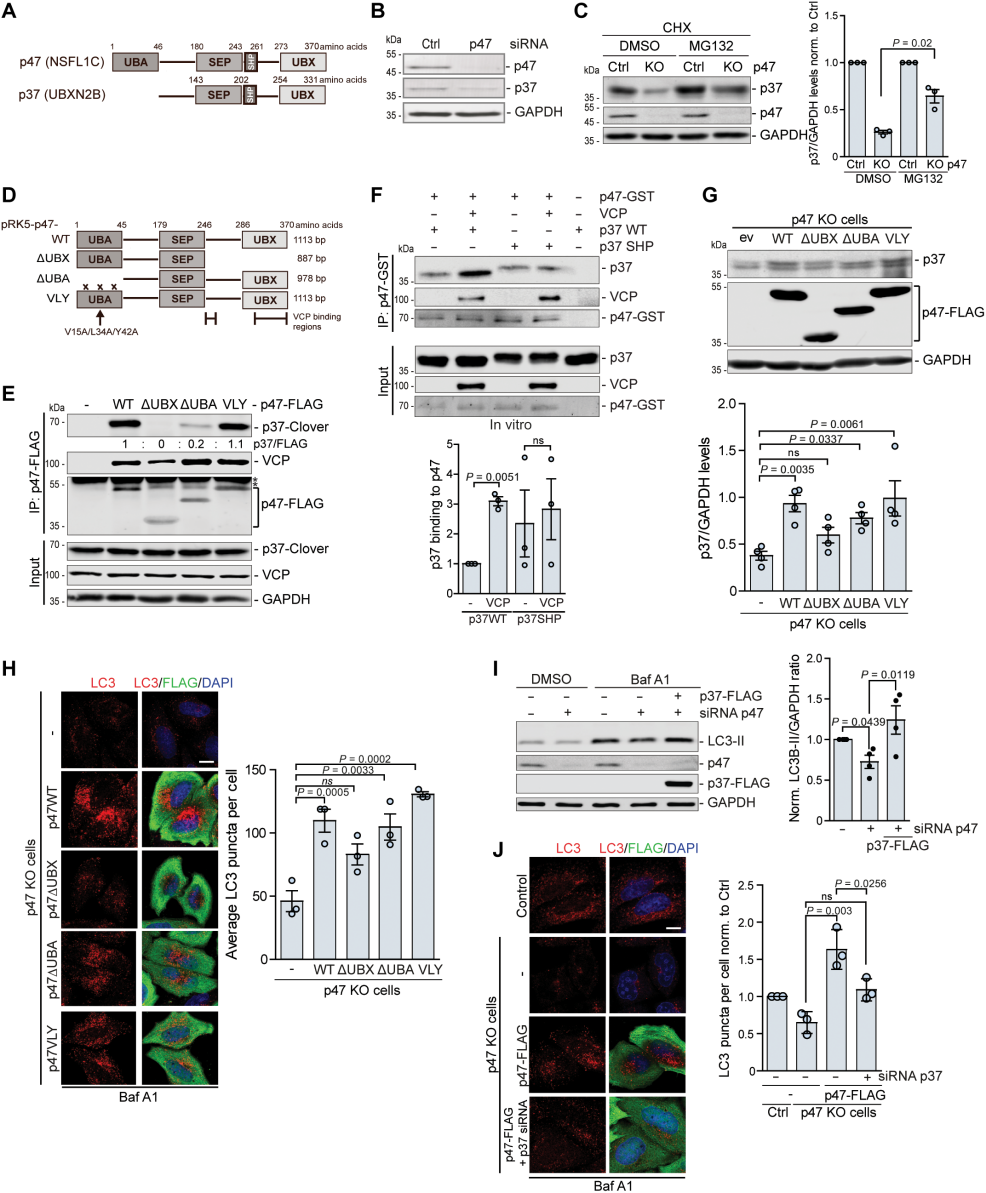
p47 regulates autophagy through direct stabilization of p37. (**A**) Domain structure of human p47 and p37. (**B** and **C**) Protein levels in HeLa cells treated with control or p47 siRNA (B) or control or p47 KO cells treated with cycloheximide (CHX) for 6 hours with 10 μM MG132 where indicated (C); *n* = 3, two-tailed paired Student’s *t* test. (**D**) Schematic representation of constructs expressing p47 mutant protein. (**E**) Immunoprecipitation of a WT and mutant p47-FLAG from HeLa cells expressing p37-Clover; *n* = 4. (**F**) In vitro binding assay. Recombinant p47-GST was immunoprecipitated together with purified WT p37, VCP nonbinding p37 SHP mutant, and VCP; *n* = 3, one-sample *t* test and two-tailed paired Student’s *t* test. (**G** and **H**) p47 WT and p47 mutants were expressed for 24 hours in p47 KO HeLa cells. (G) Western blot analysis; *n* = 4, one-way ANOVA (*P* = 0.0012) with post hoc Tukey test, p37 levels were normalized to p47-FLAG. (H) Immunocytochemistry for LC3 and FLAG after treatment with 400 nM BafA1 for 4 hours; *n* = 3, one-way ANOVA (*P* < 0.0001) with post hoc Tukey test. (**I**) Protein levels in HeLa cells treated with control or p47 siRNA for 48 hours and p37-FLAG expression for 24 hours, followed by treatment with 400 nM BafA1 for 4 hours where indicated; *n* = 4, one-sample *t* test and two-tailed paired Student’s *t* test. (**J**) p47 KO HeLa cells were treated with p37 siRNA for 48 hours and subsequent expression of p47-FLAG for 24 hours, followed by 400 nM BafA1 4-hour treatment and immunostaining for LC3 and FLAG; *n* = 3 (number of counted cells >50 per condition), one-way ANOVA (*P* = 0.0035) with post hoc Tukey test. Data are means ± SEM. Scale bars, 10 μm. *, unspecific band. In cDNA transfection experiments, matched empty vectors were used as controls for overexpression constructs, and in all knockdown experiments, we used nontargeting control siRNAs.

p47, together with p37, UBXN2A, and UBXN11 are four human homologs of yeast Shp1, which share an SEP domain and VCP-interaction module composed of the UBX domain and a SHP box that both bind to the N-terminal domain of VCP (*25*, *26*). The main structural difference between p37 and p47 is the lack of the UBA domain in the N terminus of p37 (Fig. 2A). We observed that siRNA-mediated knockdown of p47 decreased the levels of p37 (Fig. 2B). In p47 KO cells, p37 levels were also reduced in the presence of translational inhibitor cycloheximide (CHX), which was blocked by the addition of proteasome inhibitor MG132, suggesting that the absence of p47 causes more rapid proteasomal degradation of p37 (Fig. 2C).

Next, we aimed to assess the interaction between endogenous p37 and p47 by immunoprecipitation assay but did not find appropriate antibodies for this purpose, hence decided to use overexpression and in vitro approaches. p47 consists of three main domains: the N-terminal UBA domain, the central SEP domain, and the C-terminal UBX domain (Fig. 2D). To analyze the interaction between p47 and p37 and to assess which region of p47 mediates the binding with p37, we used a full-length p47 construct and p47 mutants: ∆UBX (1 to 246) lacking VCP-binding region (*25*), ∆UBA (46 to 370) lacking the UBA domain and the V15A/L34A/Y42A (VLY) mutant, which has been shown to form tight contacts with ubiquitin (*27*). We expressed p37-Clover together with FLAG-tagged full-length p47 (wild type, WT) and observed a strong interaction, which was absent for p47 ∆UBX, reduced for p47 ∆UBA, and not changed for the p47 VLY mutant (Fig. 2E). These data suggest that the UBX domain is essential for the interaction of p47 and p37, but not the UBA domain, as the VLY mutant did not affect the p47-p37 interaction and lack of the UBA domain did not completely prevent the binding. As the UBX domain is crucial for p47’s interaction with VCP, it is possible that the interaction of p47 and p37 is not direct but occurs through VCP binding or that VCP-mediates the p47-p37 interaction. To better characterize the interaction between p47 and p37, we used an in vitro approach combined with immunoprecipitation, where we incubated purified p37 with recombinant p47 protein in the presence or absence of purified VCP (Fig. 2F). We observed that p37 and p47 interact independently of VCP, but the interaction was enhanced by the presence of VCP (Fig. 2F). In addition, we used a purified p37 SHP mutant (fig. S2A), whose interaction with VCP is greatly reduced (Fig. S2B), and observed that this mutant can still interact with p47 (Fig. 2F). Similarly, when VCP was depleted from cells, the interaction between p47 and p37 was still observed (fig. S2C), further confirming that p47 can interact with p37 directly. In agreement, the expression of WT p47, p47 ∆UBA, and p47 VLY, but not p47 ∆UBX, could stabilize p37 in p47 KO cells (Fig. 2G).

Since we observed that depletion of p47 decreased the formation of autophagic structures (Fig. 1A and fig. S1B), which could be rescued by the expression of a WT p47 (fig. S1B), we expressed p47 mutants in p47 KO cells and analyzed LC3 puncta. While p47 ∆UBA and p47 VLY increased the levels of autophagosomes to a similar extent as WT p47, p47 lacking the UBX domain was less effective (Fig. 2H), correlating with the increased levels of p37 upon the expression of these p47 mutants (Fig. 2G). Furthermore, expression of p37 in p47-depleted cells rescued the formation of autophagic structures measured by the levels of LC3-II in BafA1-treated conditions (Fig. 2I) and by the number of LC3-positive structures (fig. S2D). To test directly whether p47 regulated autophagy through stabilization of p37, we expressed WT p47 in p47 KO cells pre-treated with siRNA against p37 or control siRNA. In siRNA control-p47 KO cells, p47 expression rescued the levels of p37 and numbers of LC3 puncta, but this was not seen when p37 levels were depleted (Fig. 2J and fig. S2E). Thus, p47 regulates autophagy through direct stabilization of p37 levels.

### p37 controls autophagosome biogenesis by modulating VCP association with PI3K complex I

**Fig. 3.**
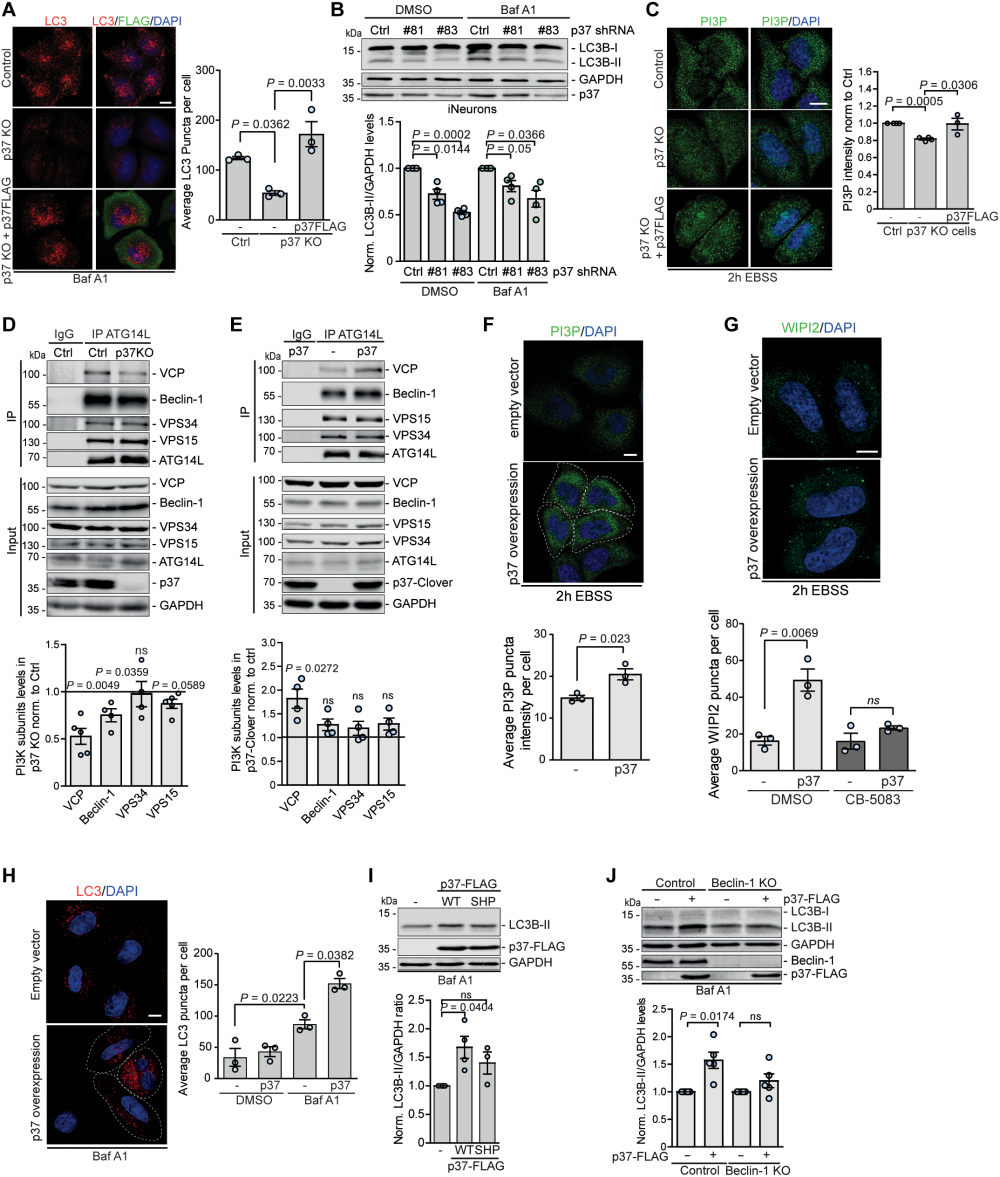
p37 regulates autophagosome biogenesis by modulating VCP association with PI3K complex I. (**A**) Control, p37 KO, and p37 KO HeLa cells reconstituted with p37-FLAG were treated with 400 nM BafA1 for 4 hours, followed by immunostaining for LC3 and FLAG; *n* = 3, one-way ANOVA (*P* = 0.004) with post hoc Tukey test. (**B**) iNeurons treated with lentiviral-delivered p37 shRNA (#81 or #83) for 4 days were treated with 400 nM BafA1 for 6 hours; *n* = 4, one-sample *t* test. (**C**) Control, p37 KO, and p37 KO with p37-FLAG–expressing HeLa cells were incubated in EBSS for 2 hours, followed by immunostaining for PI(3)P; *n* = 3 to 4, one-sample *t* test and two-tailed unpaired Student’s *t* test. (**D** and **E**) Endogenous ATG14L was immunoprecipitated from control and p37 KO HeLa cells (D) or control and p37-Clover–overexpressing HeLa cells (E); *n* = 4 to 5; one-sample *t* test. (**F** to **H**) Control or p37-FLAG–overexpressing cells were incubated in EBSS for 2 hours (F and G) and treated with 5 μM CB-5083 for 3 hours (G) or 400 nM BafA1 for 4 hours (H) where indicated, followed by immunostaining for PI(3)P (F), WIPI2 (G), or LC3 (H); *n* = 3, two-tailed unpaired Student’s *t* test. (**I**) HeLa cells expressing WT or SHP mutant p37-FLAG for 24 hours, followed by treatment with 400 nM BafA1 for 4 hours; *n* = 3 to 4, one-sample *t* test. (**J**) Control or Beclin-1 KO HeLa cells expressing p37-FLAG were treated with 400 nM BafA1 for 4 hours; *n* = 5, one-sample *t* test. Data are means ± SEM. Scale bars, 10 μm. In cDNA transfection experiments, matched empty vectors were used as controls for overexpression constructs, and in all knockdown experiments, we used nontargeting control siRNAs.

Depletion of p37 in HeLa cells by siRNA-mediated knockdown (fig. S3, A and B) or by CRISPR-Cas9–mediated KO (Fig. 3A and fig. S3C) decreased the number of autophagic structures (Fig. 3A and fig. S3B) and the levels of conjugated LC3-II in BafA1-treated conditions (fig. S3, A and C), indicating that p37 alone is important for autophagosome biogenesis. p37 was reported to be highly expressed in the human brain tissue and in neurons (*28*). We compared protein levels in several cancer cell lines, like HeLa, HEK293, and SH-SY5Y, and in induced pluripotent stem cell (iPSC)–derived human glutaminergic neurons (iNeurons) and found that the levels not only of p37 but also of VCP were much higher in neurons than in other cell types (fig. S3D), suggesting an important function of p37 in neuronal physiology. Therefore, we differentiated iPSC into mature glutaminergic neurons and decreased p37 levels using lentiviral short hairpin RNA (shRNA)–mediated knockdown with two different targeting shRNAs (#81 and #83; Fig. 3B and fig. S3E). Consistent with our data above, depletion of p37 in neurons impaired autophagosome biogenesis as measured by the decrease in the levels of LC3-II in the absence and presence of BafA1 (Fig. 3B). In line with our observations for p47 (Fig. 1, E and F), p37 depletion impaired the formation of PI(3)P and WIPI2 puncta in autophagy-inducing conditions (starvation), which could be rescued by reconstitution with WT p37 (Fig. 3C and fig. S3F), suggesting that p37 affects the activity of the PI(3)P-producing PI3K complex I.

We immunoprecipitated (IP) endogenous ATG14L from control and p37 KO cells and found that the association of VCP and Beclin-1 with the PI3K complex I was much reduced (Fig. 3D), which is known to compromise the activity of the PI(3)P producing kinase VPS34 (*10*). Conversely, overexpression of p37 significantly increased the association of VCP with ATG14L-containing PI3K complex I compared to control cells (Fig. 3E), which can account for the increased PI(3)P puncta in starvation conditions (Fig. 3F), suggesting an increase in kinase activity producing PI(3)P. In agreement, p37 overexpression increased the numbers of WIPI2 puncta, which was dependent on VCP ATPase activity as the increase was not observed when the cells were treated with the specific VCP inhibitor CB-5083 (Fig. 3G). Elevated levels of p37 increased the number of autophagic structures (Fig. 3H) and the levels of LC3-II in BafA1-treated conditions (Fig. 3I), consistent with the elevated PI3K complex I activity. The increase in LC3-II levels was dependent on the ability of p37 to effectively bind VCP (fig. S3G), as it was not observed when the p37 SHP mutant was expressed (Fig. 3I and fig. S2B). Moreover, p37-mediated autophagosome biogenesis was dependent on the presence of Beclin-1, as it was abrogated in Beclin-1 KO cells (Fig. 3J), further supporting our observations that p37 controls autophagosome biogenesis by modulating the VCP activatory effect on PI3K complex I.

### p37 regulates the clearance of misfolded and aggregate-prone proteins in the cytosol

**Fig. 4.**
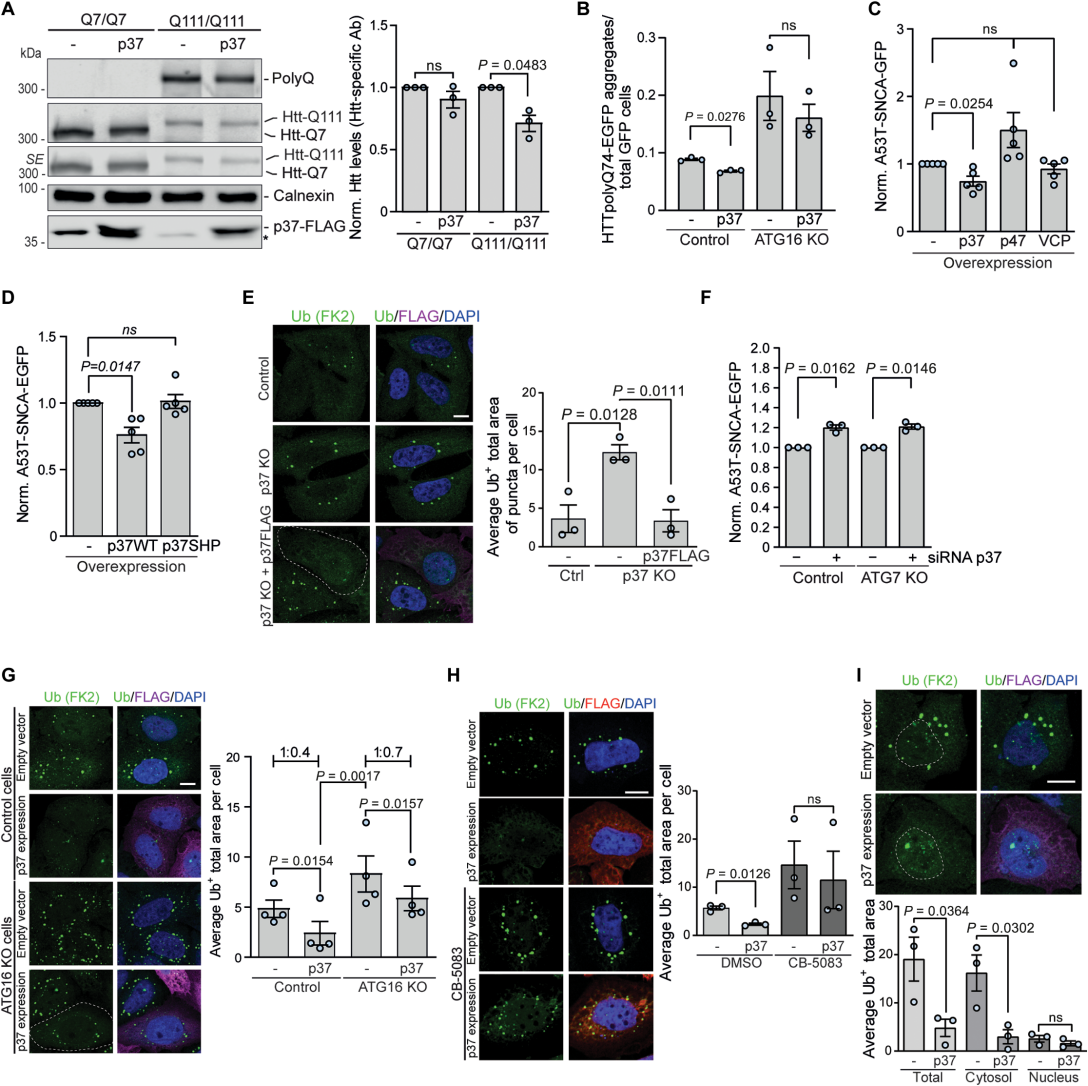
p37 regulates the clearance of misfolded and aggregate-prone proteins in the cytosol. (**A**) Mouse striatal cells with WT (Q7/Q7) or mutant (Q111/Q111) huntingtin overexpressing p37-FLAG were analyzed by western blotting; *n* = 3, one-sample *t* test. (**B**) HTTQ74-EGFP aggregates in control and p37-overexpressing cells in control or ATG16 KO HeLa cells; *n* = 3, two-tailed paired Student’s *t* test. (**C** and **D**) A53T-SNCA-EGFP HeLa cells overexpressing p37-FLAG, p47-FLAG, or VCP-HA (C) or overexpressing WT or SHP mutant p37 (D) were analyzed by FACS; *n* = 5, one-sample *t* test. (**E**) Control, p37 KO, and p37 KO expressing p37-FLAG HeLa cells were treated with puromycin for 4 hours, followed by immunostaining for ubiquitin-positive structures; quantification of the total area of ubiquitin-positive foci; *n* = 3, one-way ANOVA (*P* = 0.0074) with post hoc Tukey test. (**F**) A53T-SNCA-EGFP HeLa cells were treated with control or p37 siRNA for 48 hours, followed by FACS analysis; *n* = 3, one-sample *t* test. (**G**) Control and ATG16 KO HeLa cells overexpressing p37-FLAG were treated with puromycin for 4 hours, followed by immunostaining for ubiquitin-positive structures; quantification of the total area of ubiquitin-positive foci; *n* = 4, one-way ANOVA (*P* < 0.0001) with post hoc Tukey test. (**H**) Control and p37-FLAG–overexpressing HeLa cells pre-treated with 5 μM CB-5083 or DMSO for 1 hour were treated with puromycin for 4 hours, followed by immunostaining for ubiquitin-positive structures; quantification of the total area of ubiquitin-positive foci; *n* = 3, two-tailed paired Student’s *t* test. (**I**) Quantification of total, cytosolic, and nuclear Ub^+^ inclusions in control and p37-overexpressing cells; *n* = 3, two-tailed paired Student’s *t* test. Data are means ± SEM. Scale bars, 10 μm. *, unspecific band; SE, short exposure. In cDNA transfection experiments, matched empty vectors were used as controls for overexpression constructs, and in all knockdown experiments, we used nontargeting control siRNAs.

We previously showed that activation of VCP enhances the clearance of polyQ-expanded mutant huntingtin, the α-synuclein–A53T mutant, and misfolded protein inclusions (*11*, *29*). We confirmed that elevated p37 levels reduced the endogenous levels of mutant huntingtin in homozygous mutant huntingtin knock-in mouse striatal cell lines (Q111/Q111), but not WT huntingtin cells (Q7/Q7) (Fig. 4A). Furthermore, overexpression of p37 decreased the percentage of cells with mutant huntingtin exon 1 EGFP-HTT(Q74) aggregates in an autophagy-dependent manner, as the effect was not observed in ATG16L KO cells (Fig. 4B and fig. S4A). Furthermore, elevated expression of p37, but not p47 or VCP, decreased the levels of α-synuclein–A53T mutant protein (Fig. 4C and fig. S4B), which was dependent on the ability of p37 to bind VCP, as the effect was not observed when the p37 SHP mutant was expressed at levels comparable to WT p37 (Fig. 4D and fig. S4C). Similarly, the p37 SHP mutant failed to increase LC3-II like WT p37 (Fig. 3I).

To test the role of p37 in the degradation of misfolded proteins, we treated cells with puromycin, which incorporates into nascent chains causing premature termination of protein synthesis and release of the resulting misfolded polypeptides from ribosomes, inducing the formation of ubiquitin-positive inclusions (*11*, *30*). The lack of p37 increased the accumulation of puromycin-induced ubiquitin-positive inclusions which was rescued by the reintroduction of p37 (Fig. 4E). α-synuclein–A53T mutant protein and puromycin-induced misfolded proteins are substrates of both the ubiquitin-proteasome system and autophagy (*11*, *31*, *32*). siRNA-mediated depletion of p37 caused an accumulation of α-synuclein–A53T mutant protein in both WT and autophagy-deficient ATG7 KO cells (Fig. 4F). Moreover, p37 overexpression decreased the levels of puromycin-induced ubiquitin-positive inclusions not only in WT but also in autophagy-null cells, although to a lesser extent when compared to control cells (Fig. 4G; compare the average 1:0.4 decrease in control cells to 1:0.7 in autophagy null cells), suggesting that p37 regulates the clearance of misfolded proteins both through autophagy and UPS-dependent pathways. This effect was dependent on the VCP ATPase activity, as it was not observed in cells co-treated with the VCP inhibitor CB-5083 (Fig. 4H). The decrease in inclusion accumulation was mostly observed for cytosolic, but not nuclear puromycylated polypeptides (Fig. 4I). Thus, our data raise the possibility that elevated p37 levels promote VCP-related protein degradation in the cytoplasm, as autophagy is a cytoplasmic process.

### p37 regulates VCP shuttling between the nucleus and cytosol

**Fig. 5.**
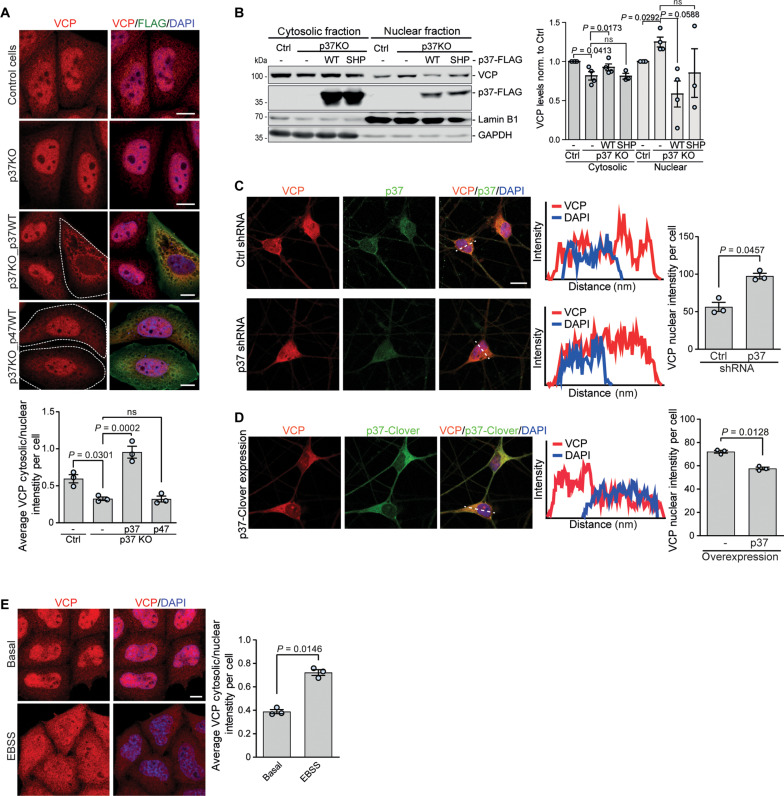
p37 levels control VCP shuttling between the nucleus and the cytosol. (**A**) Control, p37 KO, p37 KO expressing WT p37-FLAG, and p37 KO expressing WT p47-FLAG HeLa cells were immunostained for VCP and FLAG; *n* = 3, one-way ANOVA (*P* < 0.0001) with post hoc Tukey test. (**B**) Cytosolic and nuclear fractions from control, p37 KO, and p37 KO expressing WT or SHP mutant p37-FLAG HeLa cells were analyzed for VCP protein levels with Lamin B1 as a nuclear marker and GAPDH as a cytosolic marker; *n* = 4, one-sample *t* test and two-tailed paired Student’s *t* test (for nuclear p37 KO analysis). (**C** and **D**) iNeurons treated with lentiviral-delivered shRNA#81 against p37 (C) or expressing lentiviral-delivered p37-Clover (D) for 4 days were immunostained for VCP and p37; *n* = 3, two-tailed paired Student’s *t* test. (**E**) HeLa cells incubated in EBSS for 6 hours were immunostained for VCP and analyzed for VCP signal in cytosol and nucleus; *n* = 3, two-tailed paired Student’s *t* test. Data are means ± SEM. Scale bars, 10 μm. In cDNA transfection experiments, matched empty vectors were used as controls for overexpression constructs, and in all knockdown experiments, we used nontargeting control siRNAs.

VCP resides both in the cytosol and the nucleus. We observed that VCP nuclear localization is increased in p37 KO HeLa cells, and this can be reversed by the re-expression of WT p37, but not WT p47 (Fig. 5A). To confirm these findings, we isolated cytosolic and nuclear fractions from p37 KO cells transfected with empty vector, WT p37 (WT), or VCP non-binding p37 (SHP) mutant and compared the levels of VCP in each fraction to the control sample. In p37 KO cells, VCP levels were decreased in the cytosol and increased in the nucleus compared to the control cells, and this could be rescued by the expression of WT p37, but not the p37 SHP mutant (Fig. 5B). Furthermore, overexpression of WT p37, but not the p37 SHP mutant in WT HeLa cells, was sufficient to significantly decrease the levels of VCP in the nucleus (fig. S5A). Next, we depleted (Fig. 5C) or overexpressed (Fig. 5D) p37 in iNeurons and analyzed the localization of VCP. In agreement with the data above, shRNA-mediated knockdown of p37 increased VCP nuclear localization (Fig. 5C and fig. S5B) and the elevation of p37 levels resulted in nuclear depletion of VCP (Fig. 5D), indicating that the levels of p37 control the shuttling of VCP between the nucleus and the cytosol in neurons. [Because of the morphology of neurons, it is impossible to accurately assess the cytoplasmic staining of VCP. However, as p37 or p47 knockdown or KO does not change the total level of VCP, measured by Western blot analysis (see example in Fig. 1D), VCP nuclear intensity enables inference of its nucleo-cytoplasmic shuttling]. Consistent with our earlier data suggesting that p47 effects are driven by p37, we found that p47 KO, similar to p37 KO, caused nuclear relocation of VCP (fig. S5C). In p47 KO cells, this was reversed by p37 overexpression, but the nuclear localization of VCP in p37 KO cells was not affected by p47 overexpression (Fig. 5A and fig. S5C). Next, we hypothesized that in physiological conditions the shuttling of VCP between these two compartments may be regulated according to the local and acute need for VCP. We found that VCP is enriched in the cytosol and depleted from the nucleus in the autophagy-inducing starvation conditions (Earle’s balanced salt solution, EBSS) (Fig. 5E), suggesting that VCP is retained in the cytosol to fulfill its functions in the autophagy pathway. Thus, p37-regulated VCP shuttling between cytosol and nucleus may be important for cellular stress responses.

### p37-mediated VCP shuttling regulates nuclear protein degradation and cell survival upon DNA damage

**Fig. 6.**
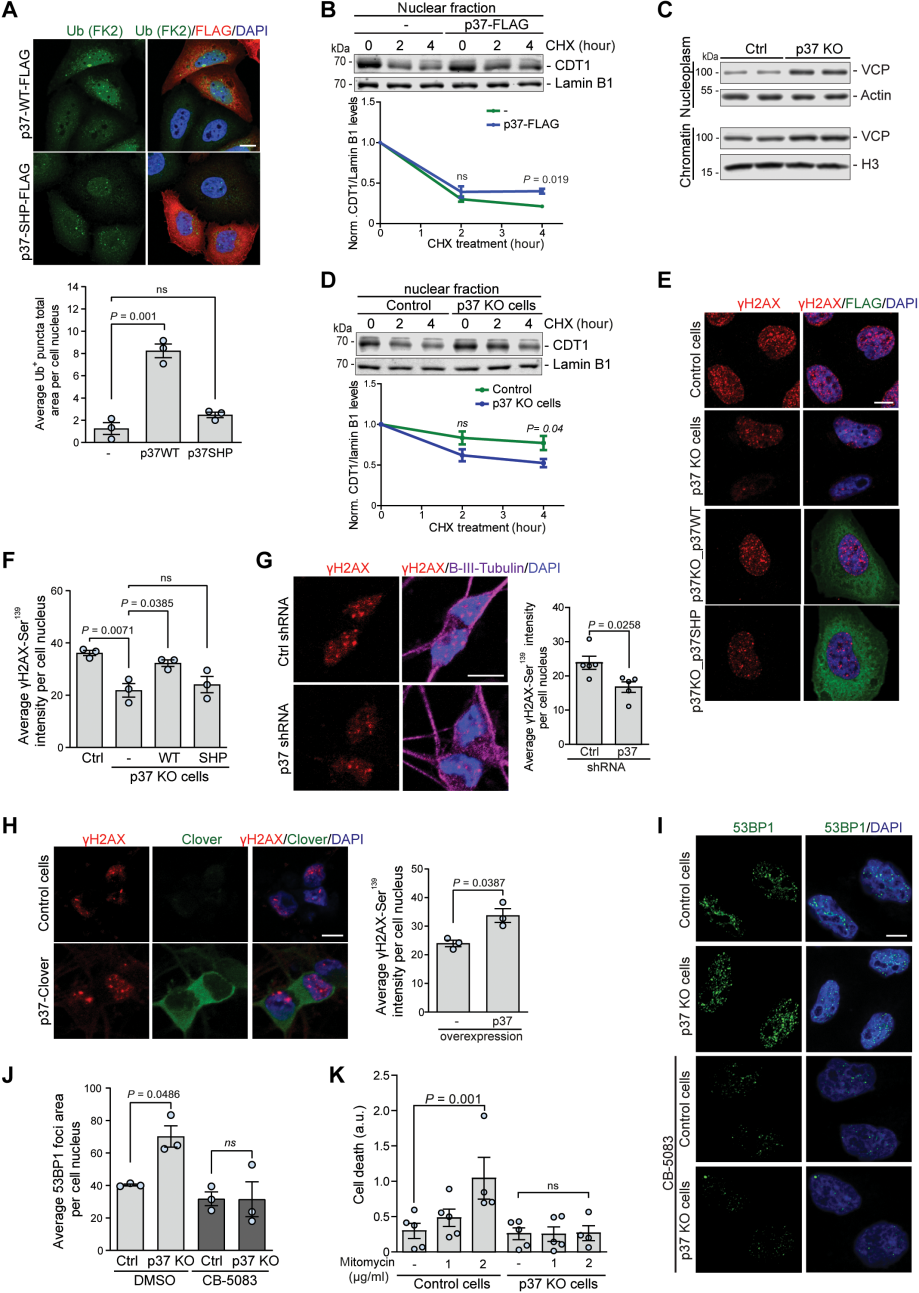
p37-mediated VCP shuttling regulates nuclear protein degradation and cell survival upon DNA damage. (**A**) HeLa cells expressing WT or SHP mutant p37-FLAG were immunostained for ubiquitin; *n* = 3, one-way ANOVA (*P* = 0.0001) with post hoc Tukey test. (**B**) Control and p37-FLAG–overexpressing HeLa cells were treated with CHX, followed by isolation of the nuclear fraction, *n* = 3, two-tailed paired Student’s *t* test. (**C**) Nucleoplasm and chromatin fraction isolated from control and p37 KO HeLa cells. (**D**) Control and p37 KO HeLa cells were treated with CHX, followed by isolation of the nuclear fraction, *n* = 3, two-tailed paired Student’s *t* test. (**E** and **F**) Control, p37 KO, and p37 KO HeLa cells expressing either WT or SHP mutant p37-FLAG were treated with mitomycin C (1 μg/ml) for 2 hours, followed by immunostaining; statistical analysis in (F); *n* = 3, one-way ANOVA (*P* = 0.0049) with post hoc Tukey test. (**G** and **H**) iNeurons treated with lentiviral-delivered shRNA#83 against p37 (G) or expressing lentiviral-delivered p37-Clover (H) were treated with mitomycin C (1 μg/ml) for 6 hours, followed by immunostaining; two-tailed paired Student’s *t* test; *n* = 5 for (G); *n* = 3 for (H). (**I** and **J**) Control and p37 KO HeLa cells treated with mitomycin C (1 μg/ml) for 2 hours in the presence or absence of 5 μM CB-5083 were immunostained for 53BP1; quantification of the total area of 53BP1 foci in (J); *n* = 3, two-tailed paired Student’s *t* test. (**K**) Control and p37 KO cells were treated with mitomycin C (1 or 2 μg/ml) for 32 hours, and cell death was monitored every 4 hours; data are represented as slope values of cell death curve over time; *n* = 4 to 5, one-way ANOVA (*P* = 0.0091) with post hoc Tukey test. Data are means ± SEM. Scale bars, 10 μm. In cDNA transfection experiments, matched empty vectors were used as controls for overexpression constructs, and in all knockdown experiments we used nontargeting control siRNAs.

VCP plays a fundamental role in genome stability by extracting ubiquitin-modified proteins from higher-order chromatin-associated complexes, thereby facilitating protein recycling, inactivation, and/or degradation by the 26*S* proteasome. Loss of VCP activity impairs the CAD pathway and leads to the accumulation of ubiquitinated substrates (*33*, *34*). We observed a significant accumulation of nuclear ubiquitin foci in cells overexpressing WT p37, but not the p37 SHP mutant (Fig. 6A), suggesting that p37-mediated depletion of VCP from the nucleus impairs the CAD pathway. During DNA replication, VCP is required for the extraction and subsequent proteasomal degradation of the DNA replication licensing factor CDT1 (*3*, *4*, *34*). We measured the dynamics of CDT1 degradation in the nucleus using CHX chase assays and found that CDT1 degradation was impaired in cells overexpressing p37 (Fig. 6B). Conversely, p37 KO cells showed an enriched VCP association with chromatin (Fig. 6C), accompanied by enhanced CDT1 degradation when compared to the control (Fig. 6D). The degradation of CDT1 was dependent on VCP ATPase activity in the p37 KO cells, as it was blocked by the VCP inhibitor CB-5083 (fig. S6A), suggesting that p37 levels may regulate the efficiency of VCP-mediated extraction and subsequent proteasomal degradation of chromatin-associated nuclear factors. In the cytoplasm, the extracting and unfolding activities of VCP facilitate proteasomal degradation of a variety of K48-linked ubiquitinated substrates. Therefore, we assessed whether increased VCP relocation into the nucleus and consequently its depletion from the cytosol in p37 KO cells affected the degradation of cytosolic VCP substrates. We monitored the degradation of K48-ubiquitinated proteins over time using a CHX chase assay and found that it was impaired in the p37 KO cells (fig. S6B). Moreover, we observed impaired clearance of the ERAD pathway substrate SCD1 (*35*) in p37 KO cells (fig. S6C) and enhanced SCD1 clearance in p37-overexpressing cells (fig. S6D), further indicating that p37-mediated regulation of VCP shuttling controls VCP local activities in both the cytosol and the nucleus.

In the nucleus, VCP plays a critical role in the DNA damage response ensuring timely and coordinated removal of recognition and repair factors, thereby facilitating DNA damage repair (*5*, *34*). We used mitomycin C to induce DNA damage that causes substantial cytotoxicity to cells and assessed the accumulation of DNA damage using γ-H2AX-Ser^319^ as a well-established marker of DNA DSBs (fig. S6E in HeLa; fig. S6F in iNeurons) (*36*). Upon mitomycin C treatment, p37 KO cells accumulated less DNA damage, monitored by the intensity of γ-H2AX staining, compared to control cells or cells where WT p37 was reintroduced (Fig. 6, E and F). Unlike overexpression of WT p37, the p37 SHP mutant that binds poorly to VCP did not increase γ-H2AX staining in p37 KO cells (Fig. 6, E and F). Furthermore, we used tert-butyl hydroperoxide (TBHP) to induce the formation of DNA single-strand breaks and monitored the accumulation of DNA damage using the alkaline comet assay. Again, the absence of p37 prevented the accumulation of DNA damage upon genotoxic stress (fig. S6G). Thus, depletion of p37, which results in elevated nuclear VCP levels, reduced DNA damage in conditions of genotoxic stress. In agreement, shRNA-mediated knockdown of p37 in iNeurons significantly decreased γ-H2AX staining (Fig. 6G), and the converse was seen with p37 overexpression (Fig. 6H). Likewise, overexpression of WT p37, but not the SHP mutant, promoted the accumulation of γ-H2AX foci in HeLa cells (fig. S6H). 53BP1 has a key role in DSB repair by promoting nonhomologous end joining (NHEJ) and its association with DSB sites is tightly controlled by VCP-UFD1L-NPL4 (*5*, *7*). We found an increase in the number of chromatin-associated 53BP1 foci in p37 KO cells compared to control (Fig. 6, I and J), which was not observed when cells were treated with the VCP inhibitor CB-5083 (Fig. 6, I and J). Theoretically, 53BP1 foci could reflect a choice of NHEJ over HR without changes in the amount of DNA damage, serve as a marker of increased DSBs, or correlate with an increased capacity to repair DNA. As we had shown that p37 depletion, which causes increased VCP nuclear localization, protects cells against DNA damage measured by either γ-H2AX or the comet assay and that p37-mediated VCP loss from the nucleus increased DNA damage measured by γ-H2AX, we believe the increased 53BP1 foci in cells with nuclear-enriched VCP reflects an increased capacity to repair DNA after damage. Mechanistically, this is consistent with previous studies that have demonstrated that the ATPase activity of VCP promotes the release of the polycomb protein L3MBTL1 from chromatin, which facilitates 53BP1 recruitment to enable DNA repair after damage (*7*). Furthermore, our assertion is supported by the observation that prolonged exposure to DNA damage agents like mitomycin C and cisplatin leads to cell death, which was ameliorated by the absence of p37 (Fig. 6K and fig. S6I for mitomycin C; fig. S6, J and K, for cisplatin). Thus, p37-mediated VCP shuttling regulates the DNA damage repair pathway and is critical for cell survival upon genotoxic stress.

### VCP proteinopathy mutation impairs DNA damage response in neurons which can be rescued by lowering p37

**Fig. 7.**
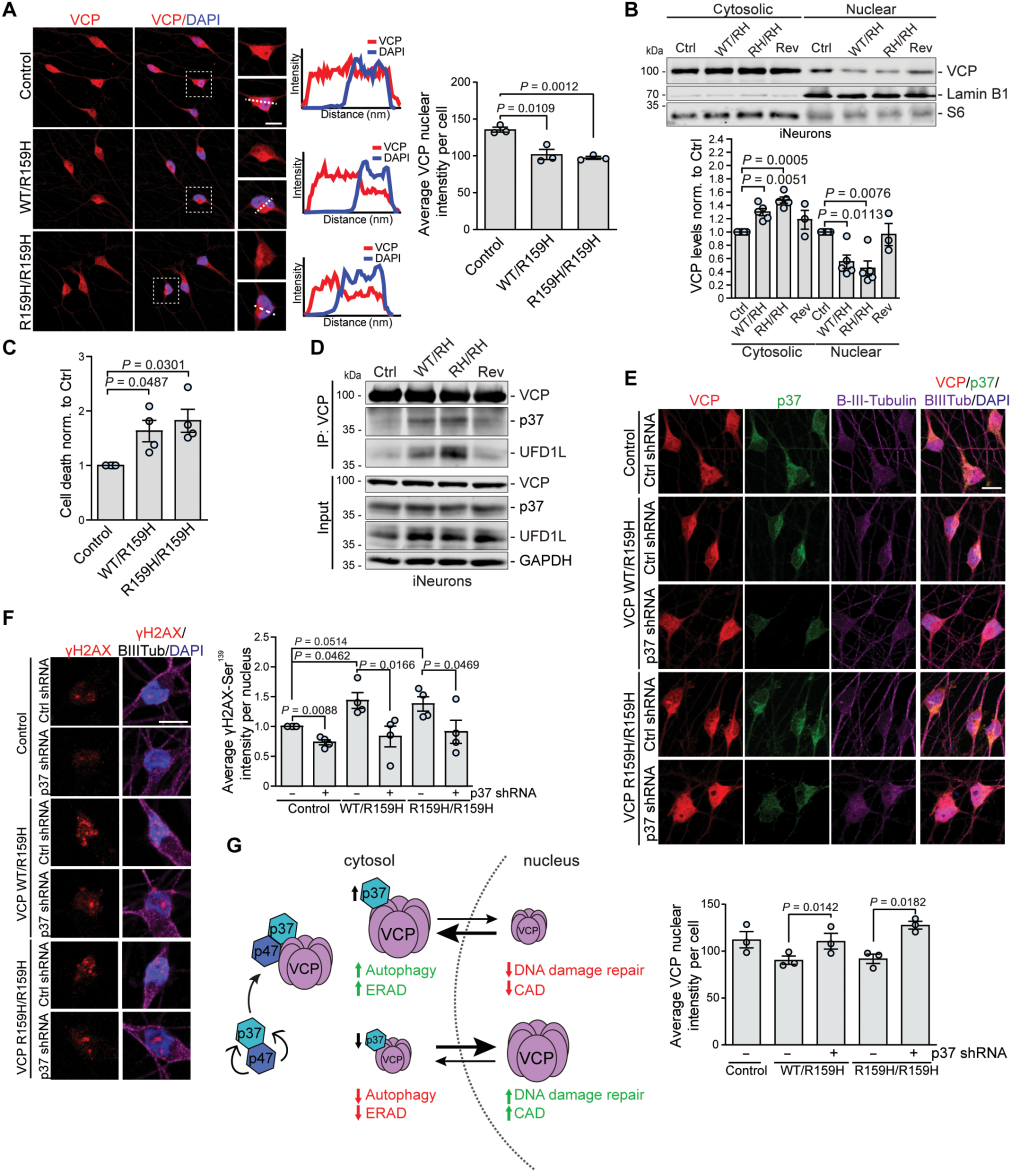
VCP R159H proteinopathy mutation impairs DNA damage response in neurons which can be rescued by the reduction in p37 levels. (**A** and **B**) Control, heterozygous VCP R159H mutant (WT/R159H), homozygous VCP R159H mutant (R159H/R159H), and control revertant (Rev) iNeurons were immunostained for VCP (A) or cytosolic and nuclear fractions were isolated (B); quantification of nuclear VCP signal in (A), *n* = 3, two-tailed paired Student’s *t* test; *n* = 3 to 5 in (B), one-sample *t* test. (**C**) Control, heterozygous VCP R159H, and homozygous VCP R159H mutant iNeurons were treated with mitomycin C (1 μg/ml) for 60 hours to measure cell death; *n* = 4; one-sample *t* test. (**D**) Endogenous immunoprecipitation of VCP from control, heterozygous VCP R159H, homozygous VCP R159H mutant, and control revertant iNeurons. (**E** and **F**) Control, heterozygous VCP R159H, and homozygous VCP R159H mutant iNeurons were treated with lentiviral-delivered control or shRNA against p37 for 4 days and immunostained; quantification of nuclear VCP signal in (E), *n* = 3; quantification of γ-H2AX-Ser^319^ intensity in (F), *n* = 4, two-tailed paired Student’s *t* test. (**G**) p37 coordinates the shuttling and local functions of VCP between the cytosol and nucleus. p47 binding to p37 prevents p37 proteasomal degradation. An increase in p37 levels leads to increased VCP localization in the cytosol, resulting in its enhanced function in ERAD and autophagy, but impaired function in CAD and DNA damage repair. Depletion of p37 promotes VCP nuclear localization and impairs VCP function in ERAD and autophagy but enhances its function in CAD and DNA damage repair. Data are means ± SEM. Scale bars, 10 μm. In cDNA transfection experiments, matched empty vectors were used as controls for overexpression constructs, and in all knockdown experiments, we used nontargeting control siRNAs.

Certain VCP disease mutations localized in the N-D1 domain of the protein, such as R155H and A232E, suppress nuclear entry of exogenously expressed VCP mutants in HEK293 cells, but the mechanism was not understood (*21*). Therefore, we differentiated human iPSCs carrying heterozygous (WT/R159H) or homozygous (R159H/R159H) disease-causing VCP R159H mutations into mature glutaminergic neurons, as confirmed by the expression of neuronal marker β-III-tubulin and the absence of Nanog, a signal for stem cells (fig. S7A). We found that both heterozygous and homozygous VCP mutant neurons had decreased VCP nuclear localization (Fig. 7, A and B) and increased VCP in the cytoplasm, when compared to the control or revertant (WT/Rev) cells (Fig. 7B). In agreement with our observations in HeLa cells, depletion of VCP from the nucleus of neurons carrying this VCP mutation sensitized cells to DNA damage stress (Fig. 7C and fig. S7B). VCP mutations leading to multisystem proteinopathy induce conformational changes in the VCP N-terminal domain resulting in alterations in the binding of VCP cofactors like UFD1L and NPL4 (*19*, *20*). We found that p37 binding to VCP was increased by the R159H (and R155H) mutations (R159H in Fig. 7D; R155H in fig. S7C), along UFD1L and NPL4. p37 binding to VCP was less tight in cells with heterozygous VCP R159H mutation, compared to homozygous cells (Fig. 7D) demonstrating that increased p37 binding is proportional to the number of mutated subunits engaged in the VCP hexameric structure. Depletion of p37 either in p37 KO HeLa cells (fig. S7D) or by shRNA-mediated knockdown in neurons (Fig. 7E) largely restored the decreased VCP nuclear localization observed in cells expressing VCP mutants, without affecting the VCP association with UFD1L-NPL4 cofactors (fig. S7E). Next, we asked whether the R159H VCP mutation and its resulting depletion from the nucleus affected the DNA damage repair pathway in human neurons. We transduced iNeurons with lentiviral vectors (expressing control shRNA and shRNA targeting p37), which, by random integration into the host genome, induce a DNA damage response. We observed that VCP mutant neurons exhibited increased γ-H2AX staining, which was rescued by p37 depletion (Fig. 7F). Thus, reduced VCP nuclear localization of VCP proteinopathy mutants impairs VCP function in the DNA damage response and this can be restored by lowering the levels of p37.

## DISCUSSION

Here, we have identified p37 as a sufficient and necessary factor regulating VCP nucleocytoplasmic shuttling. As a consequence, p37 levels fine-tune local VCP functions both in cytosolic pathways, such as autophagy and ERAD, and in the nucleus, where VCP is involved in DNA replication and DNA damage repair. Some VCP mutations causing multisystem proteinopathy increase its binding to p37 resulting in an imbalance in VCP localization, with increased levels in the cytosol and insufficient localization into the nucleus. VCP depletion from the nucleus impairs a variety of VCP nuclear functions, including in DNA replication and DNA damage repair pathways. Enhanced association between the mutant VCP and p37 is sufficient to explain the observed DNA damage repair defect in cells carrying a VCP disease-causing mutation since it could be rescued by lowering the levels of p37.

While both p47 and p37 have similar roles regulating VCP nuclear-cytoplasmic localization, autophagy, and DNA repair, our data suggest that these are driven by p37 directly and that p47 acts to stabilize p37, to which it binds. Our data suggest that a dominant role of p37 in autophagosome biogenesis is predominantly driven by the increase in cytosolic VCP, which results in the increased VCP association with PI3K complex I, enabling more PI(3)P formation. Recent studies reported that p37 facilitates VCP-mediated ubiquitin-independent biogenesis of PP1 holoenzymes by disassembling a regulatory complex of PP1 catalytic subunit with its partners SDS22 and Inhibitor-3 (*26*, *37*). Thus, it is possible that p37-VCP may additionally activate the PI3K complex I by disassembling or extracting an unknown inhibitory factor. However, more studies are needed to address this mechanistic question.

Our study revealed that p37 is a major factor regulating VCP shuttling between the nucleus and the cytosol. However, the mechanism by which p37 affects VCP shuttling remains an open question. One of the possibilities is that the p37 association modulates the exposure of two different reported N-terminal nuclear localization signals (NLS) (*38*, *39*), due to conformational changes. Point mutations in one of the NLS regions in yeast Cdc48 (*39*) or a deletion of another NLS region in human cells (*21*) were shown to suppress VCP nuclear entry. However, as alterations in the VCP N-terminal region enhance its association with cofactors, including p37, it is not clear if the effect is driven by the recognition of the VCP-NLS by nuclear import factor/s, or by enhanced retention of VCP in the cytosol due to increased p37 binding. In addition, the study in yeast reported that phosphorylation of a tyrosine residue near the C terminus correlates with the nuclear accumulation of yeast Cdc48, which was hypothesized to be caused by conformational changes leading to the exposure of the N-terminal NLS sequence (*39*). However, our data indicate that p37’s association with VCP is not likely to induce major structural changes in the N-terminal part of VCP since the binding of VCP with UFD1L and NPL4 is not changed in cells depleted of p37. In our experiments, we observed that the levels of p37 correlate with the levels of VCP retained in the cytosol, pointing to the possibility that p37 competes with the binding with an unknown factor, possibly a nuclear import factor. Further work is needed to clarify this exact mechanism.

Our work also has significance for the understanding of the cellular and systemic consequences of insufficient nuclear VCP function caused by disease-causing mutations. As VCP plays a vital role in a variety of nuclear-localized processes, including DNA replication and DNA repair, loss of the nuclear VCP early in the disease could lead to the gradual accumulation of DNA damage, an increasingly recognized and important risk factor in neurodegeneration (*40*, *41*). Accumulation of DNA damage in neuronal stem cell progenitor cells in mice with VCP multisystem proteinopathy mutation was proposed to contribute to the early stages of Frontotemporal dementia (FTD) pathology (*42*). As MSP1 (also called IBMPFD) caused by the mutations in VCP is a multisystem disease also affecting non-neuronal tissues, it raises the possibility that defects in the VCP nuclear functions may play a role in other major diseases, such as cancer. Patients with VCP disease were reported to show an increased risk of developing malignancies (*43*). Thus, increased susceptibility to DNA damage accumulation should be considered when establishing treatment strategies for patients with VCP mutations.

## MATERIALS AND METHODS

### Cell lines

Human cervical epithelium HeLa (American Type Culture Collection, #CCL-2; CVCL_0030), human embryonic kidney cell line human embryonic kidney (HEK) 293FT (Invitrogen, #R70007), SH-SY5Y (ECACC; #94030304), and striatal neuronal cell lines derived from WT HTT Q7/Q7 and homozygous HTT Q111/Q111 knock-in mice (Coriell Institute, #CH00097 and #CH00095, respectively) were cultured in Dulbecco’s modified Eagle’s medium (DMEM) [glucose (4.5 mg/liter); Sigma-Aldrich] supplemented with 10% v/v fetal bovine serum (FBS; Sigma-Aldrich), 2 mM l-glutamine (Sigma-Aldrich), and penicillin/streptomycin (100 U ml^−1^; Sigma-Aldrich).

HeLa TALEN BECLIN1 KO cell line was provided by W. Wei, Peking University, Beijing (*44*). HeLa CRISPR-Cas9 ATG16L1 KO cell line was generated using a double-nicking strategy with paired-guide RNAs and was described previously (*45*). HeLa SRAI-LC3B, EGFP–α-synuclein–A53T, and EGFP–α-synuclein–A53T ATG7 KO stable cell lines were described previously (*11*).

The G3 line of iPSCs previously derived from WTC11 was provided by M. E. Ward (*46*). Human iPSCs: parental control line (KOLF2.1 J; catalog no. JIPSC1000), VCP WT/R159H heterozygous line (SNV/WT; catalog no. JIPSC1070), VCP R159H/R159H homozygous line (SNV/SNV; catalog no. JIPSC1068), and control revertant line (REV/WT; catalog no. JIPSC1072) were purchased from Jax Laboratory.

HeLa p37 and p47 KO cell lines were generated using CRISPR-Cas9. Briefly, p37-targeting gRNA (gRNA4 ATATAGTTCGACCTTCAACTGGG) and p47-targeting gRNA (gRNA2-CGGTAGCCACCTCCTGCAAATGG) were designed using a CHOPCHOP web tool (*47*) and cloned into pKLV vector under U6 promoter [pKLV-PB-U6gRNA(BbsI)-PGKpuro2ABFP]. pKLV-U6-p37gRNA4, pKLV-U6-p47gRNA2, or a nontargeting gRNA vector control together with Cas9 expressing vector was transiently transfected into HeLa cells. Twenty-four hours after transfection, the medium was replaced with media containing puromycin (2 μg/ml) and kept for 3 days before single-cell sorting into 96-well plates using fluorescence-activated cell sorting (FACS). Clones were expanded, followed by subsequent analysis by sequencing and by SDS–polyacrylamide gel electrophoresis (PAGE) for levels of p37 or p47, accordingly.

All the cell lines (except striatal mouse cell lines which were grown at 33°C) were maintained at 37°C and 5% CO_2_ and were regularly tested for mycoplasma contamination. All cell lines were authenticated by the provider company and/or by Western blot analysis of specific proteins. For starvation experiments, cells were washed three times in starvation media EBSS (Sigma-Aldrich) and incubated for indicated time points at 37°C.

### Antibodies and reagents

The following primary antibodies have been used in this work: mouse anti-Flag M2 (catalog no. F1804, RRID:AB_262044; 1:1000), and rabbit anti-Actin (catalog no. A2066, RRID AB_476693; 1:1000) from Sigma-Aldrich; rabbit anti-VCP (catalog no. ab109240, RRID:AB_10862588; 1:2000 for WB, 1:400 for IF), rabbit anti-LC3B (catalog no. ab51520, RRID:AB_881429; 1:400 for IF), rabbit anti-GFP (catalog no. ab6556, RRID:AB_305564; 1:2000), mouse anti-GFP (catalog no. ab1218, RRID: AB_298911; 1:2000), rabbit anti-VPS15 (catalog no. ab128903, RRID: AB_11141464; 1:1000), rabbit anti-VPS34 (catalog no. ab227861, RRID: AB_2827796; 1:1000), mouse anti-GAPDH (anti–glyceraldehyde-3-phosphate dehydrogenase) (catalog no. ab8245,RRID: AB_2107448; 1:3000), rabbit anti-UFD1L (catalog no. ab96648, RRID: AB_10678868; 1:1000), rabbit anti-NPL4 (catalog no. ab101226, RRID:AB_10862595; 1:500), rabbit anti-CALNEXIN (catalog no. ab10286, RRID:AB_2069009; 1:2000), rabbit anti-ATG7 (catalog no. ab133528, RRID:AB_2532126; 1:1000), mouse anti-WIPI2 (catalog no. ab105459, RRID:AB_10860881; 1:400 for IF), mouse anti-UBXN2B (catalog no. ab124032, RRID:AB_10973365; 1:1000 for WB, 1:200 for IF), goat anti-FLAG (catalog no. 95045, RRID:AB_10676074; 1:300 for IF), rabbit anti-histone H3 (catalog no. ab18521, RRID:AB_732917; 1:1000 for WB), and rabbit anti-Nanog (catalog no. ab109250, RRID:AB_10863442; 1:400 for IF) from Abcam; rabbit anti–BECLIN 1 (catalog no. 3738, RRID: AB_490837; 1:1000), rabbit anti–K48-linkage polyubiquitin (catalog no. 8081, RRID:AB_10859893; 1:1000), rabbit anti-ATG16L1 (catalog no. 8089, RRID:AB_10950320; 1:1000), rabbit anti–phospho-p70 S6 kinase (Thr^389^; catalog no. 9234, RRID:AB_2269803; 1:1000 for WB), anti-total p70 S6 kinase (catalog no. 9202, RRID:AB_331676; 1:1000 for WB), and rabbit anti–phospho-Histone H2A.X (Ser^139^; catalog no. 2577, RRID:AB_2118010; 1:200 for IF) from Cell Signaling Technology; rabbit anti-ATG14L (catalog no. PD026, RRID: AB_1953054; 1:1000 for WB) and mouse anti-ATG14L (catalog no. M184-3, RRID: AB_10897331; for immunoprecipitation) from MBL; mouse anti-SCD1 from ATS bio (catalog no. AB-259, RRID: AB_888013; 1:1000), mouse anti-Huntingtin (catalog no. MAB2166, RRID:AB_2123255; 1:1000), and mouse anti-Polyglutamine-Expansion (catalog no. MAB1574, RRID:AB_94263; 1:1000) from Millipore; mouse anti–mono- and polyubiquitinylated conjugates (FK2) (catalog no. BML-PW8810, RRID:AB_10541840; 1:400 for IF) from EnzoLifeSciences; mouse anti-PI(3)P antibody (catalog no. Z-P003, RRID:AB_427221; 1:300 for IF) from Echelon Biosciences; mouse anti-VCP (catalog no. 60316-1-Ig, RRID:AB_2881427; 1:1000 for WB), rabbit anti-LaminB1 (catalog no. 12987-1-AP, RRID:AB_2136290; 1:1000 for WB), rabbit anti-CDT1 (catalog no. 14382-1-AP, RRID:AB_2076871; 1:1000 for WB) from Proteintech; rabbit anti-UBXN2B (catalog no. NBP1-93444, RRID:AB_11040841; 1:1000 for WB) and chicken anti-Tubulin Beta 3 (catalog no. NB100-1612, RRID:AB_10000548; 1:400 for IF) from Novus; mouse anti-53BP1 (catalog no. 612523, RRID:AB_399824; 1:200 for IF) from BD Biosciences; and rabbit anti-NSFL1C (p47) (catalog no. A304-565A, RRID:AB_2620760; 1:1000 for WB) from Bethyl.

Reagents used include BafA1 (Enzo LifeSciences, #BML-CM110), CHX (Sigma-Aldrich, #C7698), CB-5083 (Seleckchem, #S8101), SMER28 (Tocris, #4297), MG132 (Sigma-Aldrich #C2211), mitomycin C (Tocris, #3258), and cisplatin (Tocris, #2251).

### DNA constructs, siRNA, and shRNA

The following DNA constructs were used in this study: VCP(wt)-EGFP was a gift from N. Dantuma (Addgene, plasmid #23971; RRID:Addgene_23971); VCP(R155H)-EGFP was a gift from N. Dantuma (Addgene, plasmid #23972; RRID:Addgene_23972); pcDNA5FRT/TO-p37-Strep-HA was a gift from H. Meyer (Addgene, plasmid #113485; RRID:Addgene_113485); pGEX-6P-1 p37 was a gift from H. Meyer (Addgene, plasmid #113500; RRID:Addgene_113500); pGEX-6P-1 p37 SHPmut was a gift from H. Meyer (Addgene, plasmid #113503; RRID:Addgene_113503); pCW57.1 was a gift from D. Root (Addgene, plasmid #41393; RRID:Addgene_41393). pGEX-VCP-GST was shared by R. Schröder and C. Clemen (University Hospital Erlangen, Erlangen, Germany). pRK5-FLAG vector, pRK5-FLAG-p47WT, pRK5-FLAG-p47∆UBA, pRK5-FLAG-p47∆UBX, and pRK5-FLAG-p47V15A/L34A/Y42A were shared by J. Inoue (*48*) (Institute of Medical Science, University of Tokyo).

To generate the pCMV5a-p37-WT-FLAG construct, p37 was subcloned from pcDNA5FRT/TO-p37-Strep-HA into pFLAG-CMV-5a (FLAG-C-term; Sigma-Aldrich) between NotI and BamHI restriction sites using Gibson assembly method (NEB, #E2611S). To generate the pCMV5a-p37-SHP-FLAG construct, the mutation F215A/E218A/QKL220-222AAA was introduced into pCMV5a-p37-WT-FLAG using Quikchange Site-Directed mutagenesis kit (Agilent). To generate a lentiviral construct pEF-1α-p37-WT-mClover2, p37 was subcloned into the pMK1253 (gift from M. Kampmann; Addgene, plasmid #133058; RRID: Addgene_133058) between NheI and BamHI restriction sites, in frame with mClover2, using Gibson assembly method (NEB; #E2611S). To generate doxycycline-inducible pCW57.1-p37-WT-FLAG and pCW57.1-p37-SHPmut-FLAG constructs, WT and SHP mutant p37 were subcloned from pCMV5a-p37-FLAG vectors into lentiviral pCW57.1 (Addgene, plasmid #41393) between NheI and SalI restriction sites, using Gibson assembly method. All new constructs were verified by sequencing.

Pre-designed siRNAs (ON-TARGETplus SMARTpool) from Dharmacon were used as follows: control nontargeting siRNA (#D-001810-10), VCP siRNA (#L-008727-00-0005), p47 (NSFL1C) siRNA (#L-017222-01-0005), p37 (UBXN2B) siRNA (#L-025945-01-0005), ATG7 siRNA (#L-020112-00-0005), and single oligo for UBXN2B (#J-025945-13: GUGCCGUAAUAUAGAGGAAUU; #J-025945-14: GCAAAAGGUUAUACAGCCAUU; #J-025945-15: CUUCAAGAGCCCACGGACAUU; #J-025945-16: UGACUUCAUUUCCGAAUAAUU).

Simple hairpin shRNAs in the pLKO.1 lentiviral vector designed by The RNAi Consortium (TRC) were used (Horizon) as follows: TRC lentiviral empty vector control (#RHS4080), TRC lentiviral mouse Ubxn2b shRNA clone TRCN0000030081 (#RMM3981-201760388) and clone TRCN0000030083 (#RMM3981-201760390).

### Human iPSC culture and iNeuron differentiation

iPSCs were cultured in E8 Flex medium (for G3 line; Gibco, #A2858501) or in mTeSR Plus media (for VCP iPSCs lines; Stem Cell Technologies, #100-1130) on Vitronectin (Thermo Fisher Scientific) coated plates at 37°C, 5% CO_2_. iPSCs were passaged using 0.5 mM EDTA or ReLeSR solution (catalog no. 05872, STEMCELL Technologies) and maintained in up to 80% cell confluence culture with media change every second day. The Tet-ON-controlled Ngn2 transgene was introduced into VCP iPSCs lines using the PiggyBack system. Briefly, iPSC cells were transfected with PB-TO-hNGN2 (gift from iPSC Neurodegenerative Disease Initiative (iNDI) and M. Ward; Addgene, plasmid #172115; RRID:Addgene_172115) and piggyBac transposase expressing vector using Lipofectamine Stem Transfection Reagent (Thermo Fisher Scientific, catalog no. STEM00001). After 48 hours, media were replaced with mTeSR Plus media containing puromycin (4 μg/ml) to select for successfully transfected cells.

iPSCs were differentiated into glutamatergic iNeuron using a 14-day protocol. iPSCs were dissociated into single cells using Accutase (Gibco) and seeded at 250,000 cells per well onto Geltrex-coated (Gibco; #A1413302) six-well plates. On the next day, media were replaced with DMEM/F12 (Gibco, 21331-020) with N2 supplement (100×; Gibco, 17502-048), GlutaMAX (100×; Gibco, 35050-061), nonessential amino acids (100×; Gibco, 11140-035), 2-mercaptoethanol (1000x; Gibco, 31350-010), penicillin-streptomycin (100×; Gibco, 15140-122 (10,000 U/ml) with doxycycline (1 μg/ml; Sigma-Aldrich, D9891). After the pre-differentiation stage, cells were reseeded onto poly-d-lysine (Gibco, A38904-01) and Geltrex-coated (Gibco; #A1413302) plates in the Neurobasal medium (Gibco, 21103-049) with B27 supplement (50×; Gibco, 17504-044), GlutaMAX (100×; Gibco, 35050-061), 2-mercaptoethanol (1000×; Gibco, 31350-010), penicillin-streptomycin (100×; Gibco, 15140-122), NT3 (10 ng/ml; PeproTech, 45003), BDNF (10 ng/ml; PeproTech, 45002) with doxycycline (1 μg/ml). After day 7, cells were maintained in the Neurobasal growth media without doxycycline up to day 14 with half-media change every 4 to 5 days.

### Transfection

Trans IT-2020 reagent (Mirus, #MIR5400) was used for DNA transfection, while Lipofectamine 2000 (Invitrogen, #11668019) was used for siRNA transfections, according to the manufacturer’s instructions. For knockdown experiments, cells were transfected with a single round of 50 nM siRNA in Opti-MEM reduced serum media (Gibco, #31985070). Cells were harvested 2 to 3 days after transfection.

### Lentivirus production and infection

pLKO.1-shRNAs, pEF-1α-p37-WT-mClover2, and doxycycline-inducible pCW57.1-p37-WT-FLAG and pCW57.1-p37-SHPmut-FLAG containing lentiviral particles were produced and transduced following The RNAi Consortium (TRC) protocols. Briefly, HEK293FT packaging cells were transfected with a mix of packaging vector (psPAX2), envelope vector (pMD2.G), and lentiviral expression vector using Trans IT-2020 reagent (Mirus, #MIR5400) according to the manufacturer’s instructions. Transfected cells were cultured in a high-serum DMEM medium (20% FBS). Cell culture medium containing the virus was harvested every 24 hours for 3 days. Viral preps were concentrated by centrifugation at 16,100*g* for 90 min and virus particles were resuspended in 1× phosphate-buffered saline (PBS). For iPSC-derived iNeuron transduction, cells were incubated with concentrated virus overnight in the Neurobasal medium after day 14 of differentiation. On the next day, the medium was replaced with fresh medium, and cells were further incubated for 3 to 4 days before harvesting.

### Western blot analysis

To analyze protein levels, cells were lysed in RIPA buffer [150 nM NaCl, 1% NP-40, 0.5% NaDoc, 0.1% SDS, and 50 mM tris/HCl (pH 7.4)] containing protease inhibitors or directly in 1× Laemmli sample buffer [62.5 mM tris (pH 6.8), 2% w/v SDS, 10% glycerol, 50 mM dithiothreitol (DTT), and 0.01% w/v bromophenol blue], boiled for 5 min at 100°C. For Western blot analysis, samples were subjected to an SDS-PAGE and transferred on polyvinylidene difluoride (PVDF) membranes (Millipore, IPFL00005 or IPVH00005). In some experiments, after protein transfer, the PVDF membrane was cut into fragments to allow for incubation with different primary antibodies. PVDF membranes were blocked with 4% skim milk in PBS for 1 hour and incubated with primary antibodies at 4°C overnight. After washing the membrane with 0.1% Tween 20–PBS, the secondary antibodies were used at a dilution of 1:4000 and incubated for 1 hour at room temperature, followed by extensive washing with 0.1% Tween 20–PBS.

Proteins on the membrane were visualized with direct infrared fluorescence detection on a LI-COR Odyssey scanner. Densitometric analysis on the immunoblots was performed using IMAGE STUDIO Lite software, which enables quantitative analysis of blotting signals.

### Immunofluorescence and image analysis

For imaging, HeLa cells were fixed in paraformaldehyde 4% for 10 min and neurons were fixed in paraformaldehyde with 4% sucrose. Fixed cells were permeabilized with 0.1 to 0.2% Triton X-100 for 5 min, blocked in 1% BSA for 45 min at room temperature, followed by incubation with indicated primary antibodies for 2 hours at room temperature, and with secondary Alexa Fluor antibodies for 1 hour at room temperature. For imaging of LC3 puncta, cells were fixed in ice-cold methanol for 5 min, blocked in 1% BSA at room temperature for 1 hour, and then incubated with rabbit anti-LC3B (Abcam, #ab192890) for 1 hour at room temperature, and with secondary Alexa Fluor for 1 hour at room temperature. Stained cells were washed three times with 1× PBS and mounted on microscope slides with ProLong Gold Antifade Mountant with DAPI (Thermo Fisher Scientific).

The staining of PI(3)P was performed as described previously (*10*, *49*). Briefly, cells were fixed in 2% w/v paraformaldehyde, permeabilized with 20 μM digitonin, and blocked with 5% v/v FBS. Mouse anti-PI(3)P antibody (1:300; 1 hour at room temperature) (Echelon Biosciences catalog no. Z-P003, RRID:AB_427221) and secondary antibody (1:400; 30 min at room temperature) (goat anti-mouse Alexa Fluor 555) were applied, followed by post-fixation in 2% paraformaldehyde, washing and mounting on microscope slides with ProLong Gold Antifade Mountant with DAPI (Thermo Fisher Scientific). Imaging was conducted with LSM710 or LSM880 Zeiss confocal with a 63× oil-immersion lens.

Analyses of the number of puncta (LC3 and WIPI2) or total area of the puncta (Ubiquitin and 53BP1) were performed in ImageJ, with manual annotation of cell boundaries using region of interest (ROI) and automatic analysis of the number of puncta or total area of puncta per cell using particle analysis plugin, using the same cutoff for puncta identification in all conditions. White dashed circle lines on representative images indicate cells that were positively stained for overexpressed protein (FLAG-tagged p37 or p47). In Fig. 4I, white dashed circle lines indicate the boundaries of the nucleus. Signal intensity (γ-H2AX, VCP, and GFP) analysis was performed in ImageJ with manual annotation of cell boundaries using ROI and automatic measurement of signal intensity. A minimum of 30 cells were examined for each condition. All experiments were repeated in at least three biological replicates. Images were analyzed using ZEN Black Carl Zeiss Microscopy.

### Cytotoxicity measurement

Cytotoxicity of DNA damage-inducing factors was assessed using CellTox Green Dye (Promega, #G8741) where cell death was monitored in real time using Incucyte live cell analysis (Essen Bioscience, IncuCyte S3 with ×10 magnification). Briefly, for HeLa cell experiments, cells were seeded into a 96-well plate and mitomycin or cisplatin were added at the indicated concentrations together with CellTox Green Dye in 1:1000 dilution at time 0. For iNeurons experiments, pre-differentiated neuronal cells were seeded into 24-well plates and differentiated up to day 12. On day 12, cisplatin was added together with CellTox Green Dye in 1:1000 dilution at time 0, and green fluorescence was measured starting from 20 hours after treatment. Cells were cultured in the Incucyte instrument, and the green fluorescence (Ex: 450 to 490 nm; Em: 500 to 530 nm) was recorded with five fields per well. Cell death manifesting with green fluorescence was normalized to the total phase area of cells at each indicated time point. Data are represented and statistical analysis was performed using slope values of detected increase in toxicity for each sample over a period of up to 32 hours for HeLa cells and up to 60 hours for iNeurons.

### Nuclear and cytoplasmic fractionation

For nuclear and cytoplasmic fractionation of HeLa cells, cells grown on 100-mm dishes were washed with 1× PBS and lysed in 500 μl of lysis buffer A [20 mM tris (pH 7.4), 10 mM KCl, 0.1 mM EDTA, 0.5% NP-40, and protease inhibitors cocktail] for 30 min on ice. Lysates were centrifuged for 10 min at 16,000*g* at 4°C and supernatants were collected as cytosolic fractions. The remaining pellets were washed once with lysis buffer A and resuspended in 150 μl of lysis buffer B [20 mM tris (pH 7.4), 400 mM NaCl, 1 mM EDTA, 10% glycerol, and protease inhibitors cocktail] for 1 hour on ice. Lysates were centrifuged for 10 min at 16,000*g* at 4°C and supernatants were collected as nuclear fractions. Laemmli sample buffer (4×) was added to both fractions and samples were boiled for 5 min at 100°C and analyzed by Western blot. Nucleoplasm and chromatin isolation in HeLa cells was performed according to the published protocol (*50*).

For nuclear and cytosolic fractionation of iNeurons, cells were grown on a 60-mm dish washed with 1× PBS and trypsinized by adding 500 μl of Accutase and incubation at 37°C for 3 min. The activity of Accutase was subsequently inhibited by the addition of Neurobasal medium. Collected cells were washed once with 1× PBS, resuspended in 180 μl of lysis buffer A [50 mM Hepes (pH 7.4), 150 mM NaCl, digitonin (25 μg/ml), 1 M hexylene glycol, and protease inhibitors cocktail] and incubated rotating for 10 min at 4°C. Lysate was centrifuged at 2000*g* for 10 min at 4°C and supernatant was collected as a cytosolic fraction. The remaining pellet was resuspended in 180 μl of lysis buffer B [50 mM Hepes (pH 7.4), 150 mM NaCl, 1% NP-40, 1 M hexylene glycol, and protease inhibitors cocktail] and incubated for 30 min on ice, followed by centrifugation at 7000*g* for 10 min at 4°C. The supernatant was discarded, and the remaining pellet was resuspended in 180 μl of lysis buffer C [50 mM Hepes (pH 7.4), 150 mM NaCl, 0.5% sodium deoxycholate, 0.1% SDS, 1 M hexylene glycol, protease inhibitors cocktail, and Benzonase] and incubated rotating for 30 min at room temperature, followed by centrifugation at 8000*g* for 10 min at 4°C. The supernatant was collected as a nuclear fraction, 4× Laemmli sample buffer was added to both fractions, and samples were boiled for 5 min at 100°C and analyzed by Western blot.

### Alkaline comet assay

To assess both single and double-strand DNA breaks, HeLa cells were treated for 1 hour with 100 μM TBHP before comet assay. Cells were harvested by trypsinization and resuspended in ice-cold 1× PBS. Around 1500 cells were gently mixed with 60 μl of 1% low-melting agarose (LMA; Bio-Techne Ltd. 4250-050-02), and then placed onto microscope slides precoated with 1% normal melting agarose. Coverslips (15 mm × 15 mm) were immediately placed onto cell/LMA mixtures to cast them into a circular agarose gel. Slides were left at 4°C for 10 min before removing the coverslip and were subsequently lysed for 90 min in lysis buffer [2.5 M NaCl, 0.1 M EDTA, 10 mM tris (pH 10), freshly supplemented with 10% dimethyl sulfoxide and 1% Triton X-100]. After lysis, slides were left in fresh alkaline unwinding buffer (0.3 M NaOH and 1 mM EDTA) for 30 min followed by electrophoresis at 1 V/cm, 300-mA electric field, in the same buffer. After electrophoresis, slides were washed with ddH_2_O twice and once with 70% ethanol and lastly dried overnight. Nucleic acid staining was performed the next day with SYBR Green (Invitrogen, S7563). Samples were imaged with AxioObserver and the results were analyzed using the OpenComet plugin in Fiji ImageJ. Data are presented as a percentage of tail comet DNA content and as Olive Moment (product of tail DNA% and the distance between the intensity-weighted centroids of head and tail).

### Protein purification

Purification of GST-VCP, p37 WT, and p37 SHP mutant from *Escherichia coli* was performed as described previously (*10*). Recombinant p47-GST purified from wheat germ was obtained commercially (Novus, H00055968-P01). Briefly, the expression vector was transformed into the bacterial strain Rosetta 2 BL21 (DE3) (Novagen) according to instructions from the supplier. Cells from a 1-liter culture were harvested after induction of protein expression with 0.2 mM isopropyl-β-d-thiogalactopyranoside (IPTG) at 18°C for 18 hours for VCP protein and 0.4 mM IPTG at 37°C for 4 hours for p37. The cell pellet was resuspended in lysis buffer (2× PBS, 20 mM MgCl_2_ for VCP containing protease inhibitors and lysed by incubation with lysozyme (0.5 mg/ml) and deoxyribonuclease I (1 U/ml) for 30 min on ice, followed by sonication. Lysates were clarified by ultracentrifugation (100,000*g* for 20 min at 4°C). Clarified lysates were incubated with 1 ml of glutathione sepharose resin (GST, Pierce) for 2 hours at 4°C. The resin was added to the gravity flow column and washed with wash buffer (lysis buffer and 0.1% Triton X-100), followed by three 5-min washes with washing buffer containing 1 mM ATP. VCP and p37 proteins were eluted from glutathione beads by cleaving the GST tag with PreScission protease. For removal of the GST tag, beads were washed five times with PreScission cleavage buffer [50 mM tris (pH 7.0), 150 mM NaCl, 1 mM EDTA, 1 mM DTT, and 0.01% Triton X-100] and resuspended in 500 μl of cleavage buffer containing PreScission protease (80 U/ml), incubated overnight at 4°C, followed by collection of supernatant containing purified protein. Purified proteins were analyzed on SDS-PAGE gel stained with Coomassie blue to determine sample purity and concentration against the BSA dilution curve.

### In vitro binding assay

VCP, p37WT, and p37SHP were purified from *E. coli*, and recombinant p47-GST was obtained commercially (Novus, H00055968-P01). Empty glutathione beads were used as a control and the buffer composition and incubation conditions were optimized to minimize the interaction of non-GST-tagged proteins with empty beads (p37WT). Two micrograms of VCP and p37 protein was used per reaction with 1 μg of recombinant p47. Proteins were incubated in binding buffer [25 mM Hepes (pH 7.4), 150 mM NaCl, 0.01% Triton X-100, and 5% glycerol] for 1 hour rotating at 4°C without the beads, followed by 1-hour incubation with 20 μl of pre-washed glutathione beads. Input samples were collected before the addition of beads. Next, protein-loaded GST beads were washed three times with binding buffer, followed by protein elution with 2× Laemmli sample buffer for 5 min at 100°C.

### Immunoprecipitation

For immunoprecipitation of endogenous proteins (ATG14L or VCP), cells grown on 150-cm^2^ dishes were washed with 1× PBS and lysed in 1 ml of IP buffer [for ATG14L IP: 20 mM tris (pH 7.4), 2 mM MgCl_2_, 200 mM NaCl, 0.5% NP-40, and protease inhibitors cocktail; for VCP IP: 50 mM tris (pH 7.4), 2 mM MgCl_2_, 150 mM NaCl, 0.5% NP-40, 10% glycerol, and protease inhibitors cocktail] for 30 min on ice, followed by centrifugation for 10 min at 20,000*g* at 4°C. Input samples were collected and the rest of the supernatant was incubated with primary antibodies rotating overnight at 4°C, followed by the addition of 20 μl of pre-washed magnetic Dynabeads Protein G (Invitrogen) and incubation for further 2 hours at 4°C. Protein-loaded beads were washed three times with lysis buffer without detergent and proteins were eluted in 2× Laemmli sample buffer by boiling and analyzed by Western blot.

For immunoprecipitation of overexpressed VCP-EGFP or FLAG-tagged proteins, cells grown on 55-cm^2^ dishes were washed with 1× PBS and lysed in 1 ml of IP buffer [50 mM tris (pH 7.4), 2 mM MgCl_2_, 150 mM NaCl, 0.5% NP-40, 10% glycerol, and protease inhibitors cocktail] for 30 min on ice, followed by centrifugation for 10 min at 20,000*g* at 4°C. Input samples were collected and the rest of the supernatant was incubated with 6 μl of GFP-Trap Magnetic Agarose (ChromoTek) or 5 μl of Anti-FLAG M2 Magnetic Beads (Millipore, #M8823) for 1 hour rotating at 4°C, followed by three washes with lysis buffer without detergent and elution in 2× Laemmli sample buffer by boiling and analyzed by Western blot.

### FACS analysis of mutant α-synuclein (A53T) and SRAI-LC3B

HeLa cells stably expressing GFP-tagged mutant α-synuclein (EGFP-A53T) were treated with siRNA for 48 to 72 hours before analysis. Cells were then trypsinized and GFP fluorescence was analyzed using an Attune NxT Flow Cytometer (Thermo Fisher Scientific) using the BL1 (488 530/30) detector. Cells were first gated on forward (FSC-A) and side scatter (SSC-A) for P1 and then for singlets (FSC-A/FSC-H) for P2. A total of 20,000 single cells were recorded for each replicate. GFP^+^ gates were set using normal unstained HeLa cells. HeLa cells stably expressing SRAI-LC3B were treated with siRNA for 48 to 96 hours before analysis and with SMER28 where indicated. Cells were trypsinized and analyzed using an Attune NxT Flow Cytometer (Thermo Fisher Scientific) using the VL2 (405 512/25) and BL1 (488 530/30) detectors. The ratio of VL2 to BL1 signals was derived for each cell and the median ratio per condition was used for analysis. The data were analyzed using FlowJo software v10.7.1.

### Statistical analysis

Significance levels for comparisons between groups were determined using GraphPad Prism versions 7 and 8 (GraphPad Software) or Excel (Microsoft Office). For Western blots, protein levels were normalized to a housekeeping protein, such as actin, GAPDH, or tubulin. In cell fractionation experiments, protein levels were normalized to cytosolic markers, such as GAPDH or S6, or to nuclear marker Lamin B1. For immunostaining, in experiments where cells were transfected with FLAG-tagged proteins (such as p37 or p47-FLAG WT and mutants), only cells positive for FLAG signal were analyzed. In experiments where we have scored the percentage of cells/nuclei showing a discrete phenomenon, we have assessed at least 50 cells per individual experiment in cell lines or at least 30 cells in neurons. In each experiment where gamma H2AX staining was assessed in HeLa cells, we scored around 100 nuclei per condition (ranging from 78 to 130), and figures presented in the paper show statistics of mean data from at least three independent biological replicates (around 300 nuclei quantified per condition in total). All figures show statistics of means from immunostaining experiments from at least three biological replicates. Error bars shown in the figures represent an SEM. *P* < 0.05 was considered statistically significant. Statistical analysis was performed using one- or two-tailed Student’s *t* test, one-sample *t* test, or one-way analysis of variance (ANOVA) followed by an appropriate post hoc test for multiple comparisons. Sample sizes were chosen on the basis of extensive experience with the assay performed. Each experiment was repeated at least three times as an independent biological replicate. The experiments were appropriately randomized and blinded when possible.
