# Supplementary material for: p37 regulates VCP/p97 shuttling and functions in the nucleus and cytosol
Source: Sci Adv. 2024 May 3;10(18):eadl6082. doi: 10.1126/sciadv.adl6082 (PMC11068011; doi:10.1126/sciadv.adl6082)
Supplement: Supplementary file 1 — Figs. S1 to S7 [file sciadv.adl6082_sm.pdf]

Science Advances
AAAS

Supplementary Materials for

p37 regulates VCP/p97 shuttling and functions in the nucleus and cytosol

Lidia Wrobel *et al.*

Corresponding author: David C. Rubinsztein, dcr1000@cam.ac.uk

Sci. Adv. **10**, eadl6082 (2024)
DOI: 10.1126/sciadv.adl6082

This PDF file includes:

Figs. S1 to S7

Figure S1

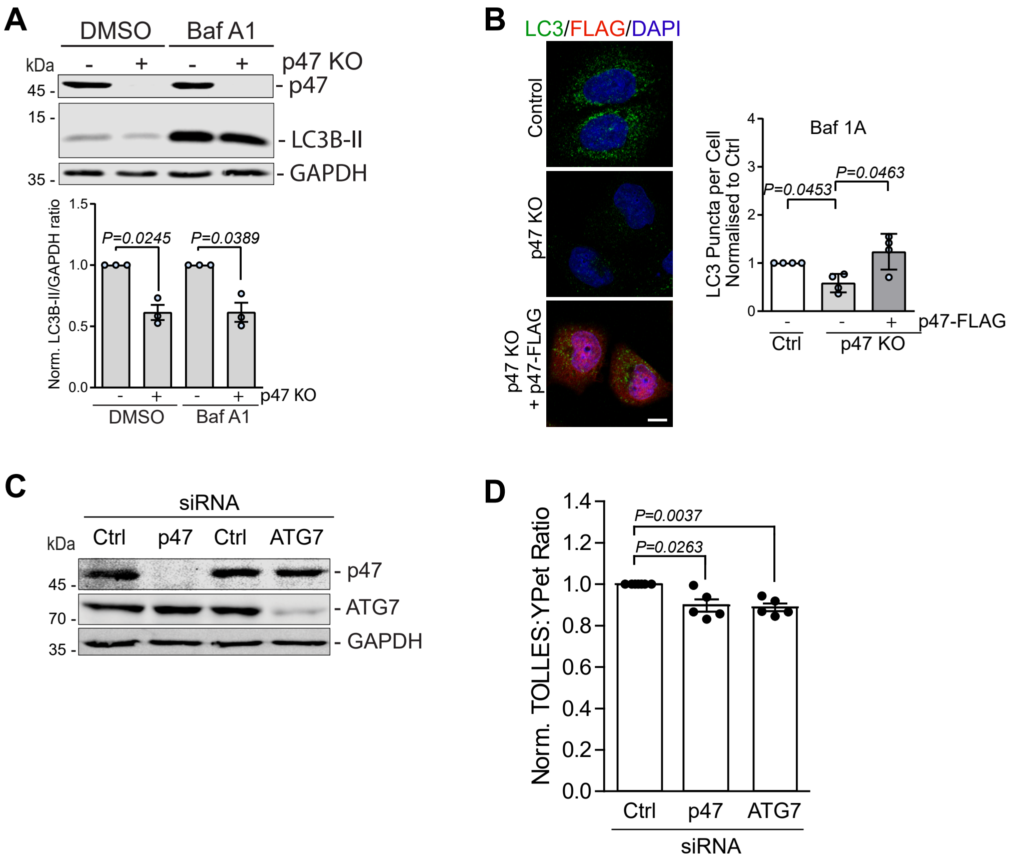
A
DMSO
Baf A1
kDa
p47 KO
45 -
- p47
15 -
- LC3B-II
35 -
- GAPDH
Norm. LC3B-II/GAPDH ratio
P=0.0245
P=0.0389
p47 KO
DMSO
Baf A1
B
LC3/FLAG/DAPI
Control
p47 KO
p47 KO + p47-FLAG
LC3 Puncta per Cell Normalised to Ctrl
Baf 1A
P=0.0453
P=0.0463
p47-FLAG
Ctrl
p47 KO
C
siRNA
kDa
Ctrl
p47
Ctrl
ATG7
45 -
- p47
70 -
- ATG7
35 -
- GAPDH
D
Norm. TOLLES:YPet Ratio
P=0.0037
P=0.0263
Ctrl
p47
ATG7
siRNA

fig. S1. p47 regulates autophagy.

(**A**) Control and p47 knockout HeLa cells were treated with 400 nM Bafilomycin A1 (BafA1) for 4 h, followed by western blot analysis; n=3, one sample t-test, data are mean ± SEM. (**B**) Control, p47 knockout and p47 knockout expressing wild-type p47-FLAG HeLa cells were treated with 400 nM BafA1 for 4 h, followed by immunostaining for LC3 puncta and FLAG; quantification of LC3 puncta normalised to control (n=4, one-way ANOVA (P=0.0296) with post hoc Tukey test), data are mean ± SEM. (**C**) Protein levels were analysed in HeLa cells expressing SRAI-LC3B treated with control or siRNA against p47 or ATG7. Related to Fig. 1G and Fig. S1E. (**D**) HeLa cells expressing SRAI-LC3B treated with control or siRNA against p47 or ATG7 were analysed by FACS; n=5, one sample t-test, data are mean ± SEM. Scale bars, 10 μm. In all cDNA transfection experiments, matched empty vectors were used as controls for overexpression constructs. Similarly, in all knockdown experiments we used non-targetting control siRNAs.

Figure S2

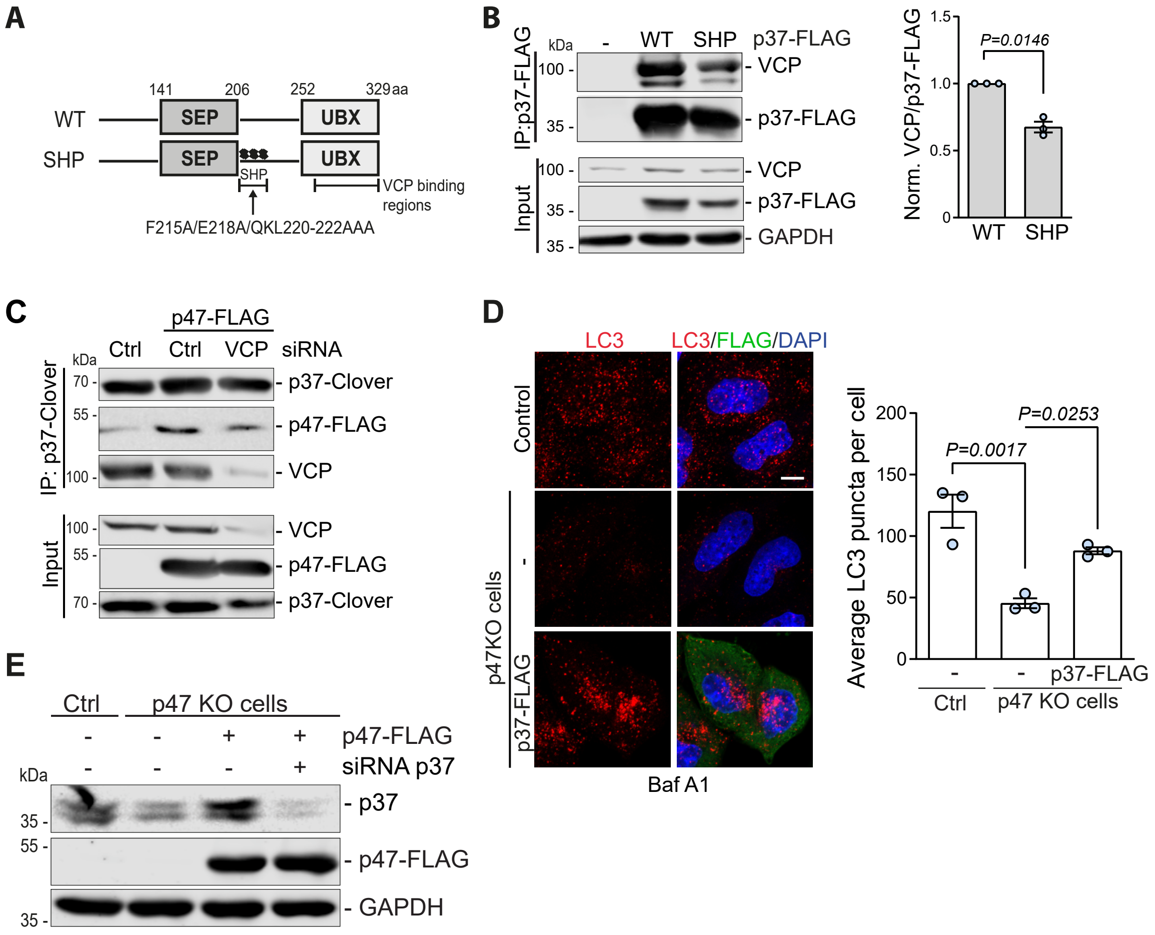

A
141
206
252
329aa
WT
SEP
UBX
SHP
SEP
UBX
SHP
VCP binding regions
F215A/E218A/QKL220-222AAA
B
kDa
-
WT
SHP
p37-FLAG
IP:p37-FLAG
100 -
- VCP
35 -
- p37-FLAG
Input
100 -
- VCP
35 -
- p37-FLAG
35 -
- GAPDH
Norm. VCP/p37-FLAG
P=0.0146
1.5
1.0
0.5
0
WT
SHP
C
p47-FLAG
Ctrl
Ctrl
VCP
siRNA
kDa
IP: p37-Clover
70 -
- p37-Clover
55 -
- p47-FLAG
100 -
- VCP
Input
100 -
- VCP
55 -
- p47-FLAG
70 -
- p37-Clover
D
LC3
LC3/FLAG/DAPI
Control
p47KO cells
-
p37-FLAG
Baf A1
Average LC3 puncta per cell
P=0.0017
P=0.0253
200
150
100
50
0
-
-
p37-FLAG
Ctrl
p47 KO cells
E
Ctrl
p47 KO cells
-
-
+
+
p47-FLAG
-
-
-
+
siRNA p37
kDa
35 -
- p37
55 -
- p47-FLAG
35 -
- GAPDH

fig. S2. p47 regulates p37 levels.

(**A**) Schematic representation of constructs expressing wild-type (WT) p37 and p37 SHP mutant. (**B**) Immunoprecipitation (IP) of a wild-type (WT) p37 and p37 SHPmutant-FLAG from HeLa cells. Binding between VCP and p37 SHP mutant was decreased when compared to wild-type p37; n=3, one sample t-test. (**C**) Immunoprecipitation (IP) of wild-type p47-FLAG from HeLa cells expressing wild-type p37-Clover. Cells were pretreated with siRNA against VCP for 48 h prior IP where indicated. Interaction between p47 and p37 was still observed in cells were VCP was depleted. (**D**) Control, p47 knockout and p47 knockout cells expressing wild-type p37-FLAG were treated with 400 nM Bafilomycin A1 (BafA1) for 4 h, followed by immunostaining for LC3 puncta and FLAG; n=3, one-way ANOVA (P=0.0021) with post hoc Tukey test. (**E**) Wild-type p47-FLAG was expressed in p47 knockout HeLa cells for 24 h, pre-treated with siRNA against p37 for 72 h where indicated, followed by western blot analysis together with control cells. Data are mean ± SEM, scale bars, 10 μm. In all cDNA transfection experiments, matched empty vectors were used as controls for overexpression constructs. Similarly, in all knockdown experiments we used non-targetting control siRNAs.

Figure S3

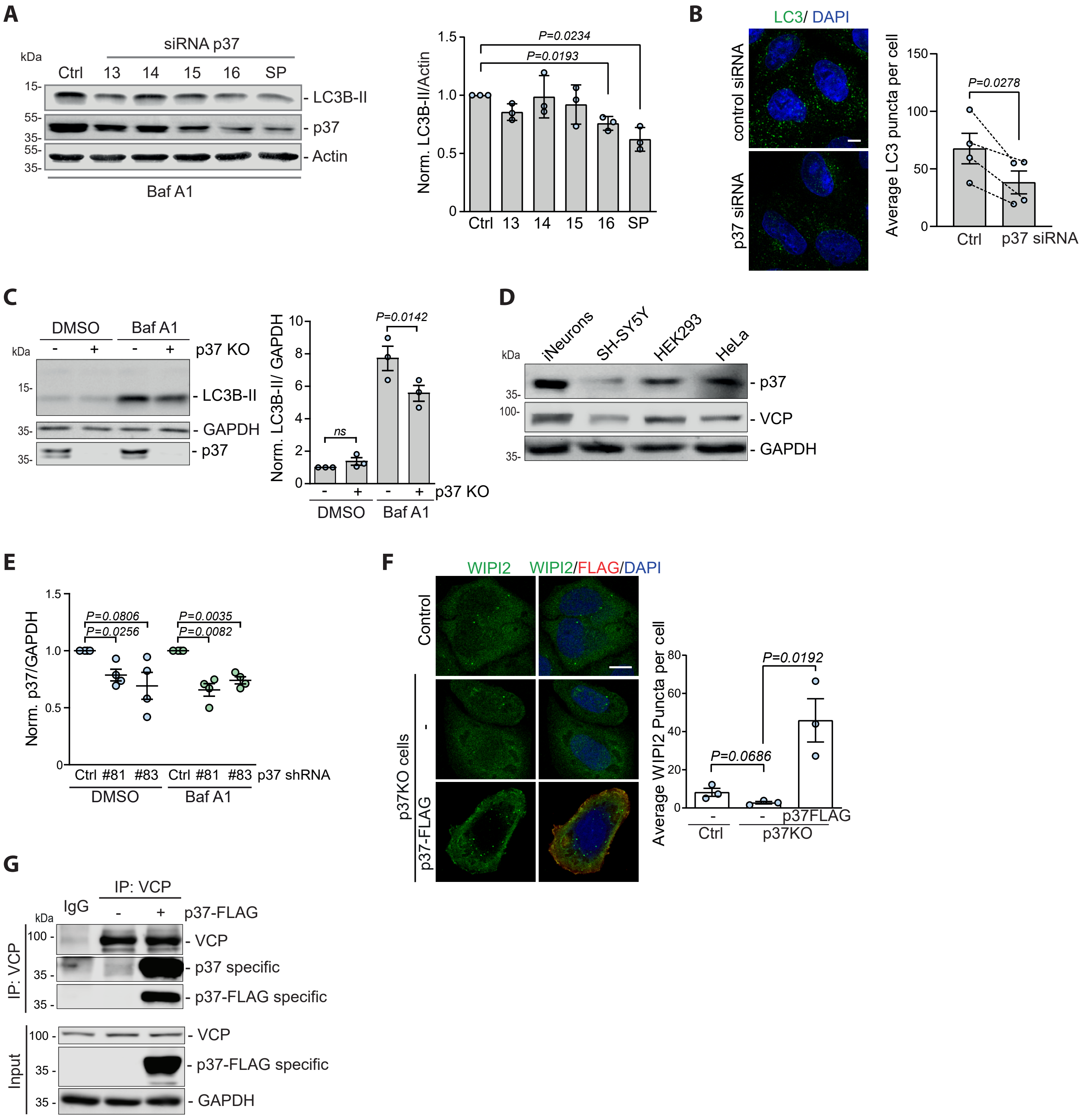
A
siRNA p37
kDa
Ctrl 13 14 15 16 SP
15-
55-
35-
55-
35-
- LC3B-II
- p37
- Actin
Baf A1
Norm. LC3B-II/Actin
P=0.0234
P=0.0193
Ctrl 13 14 15 16 SP
B
LC3/ DAPI
control siRNA
p37 siRNA
Average LC3 puncta per cell
P=0.0278
Ctrl p37 siRNA
C
DMSO Baf A1
p37 KO
- LC3B-II
- GAPDH
- p37
Norm. LC3B-II/ GAPDH
P=0.0142
ns
DMSO Baf A1
D
iNeurons SH-SY5Y HEK293 HeLa
- p37
- VCP
- GAPDH
E
Norm. p37/GAPDH
P=0.0806
P=0.0256
P=0.0035
P=0.0082
Ctrl #81 #83 Ctrl #81 #83 p37 shRNA
DMSO Baf A1
F
WIPI2 WIPI2/FLAG/DAPI
Control
p37KO cells
p37-FLAG
Average WIPI2 Puncta per cell
P=0.0192
P=0.0686
p37FLAG
Ctrl p37KO
G
IgG IP: VCP
p37-FLAG
- VCP
- p37 specific
- p37-FLAG specific
IP: VCP
Input
- VCP
- p37-FLAG specific
- GAPDH

Fig. S3. p37 regulates autophagosome biogenesis.

(**A**) HeLa cells were treated with control, smart pool (SP) or single siRNA oligos against p37 for 48 h, followed by treatment with 400 nM Bafilomycin A1 (BafA1) for 4 h and western blot analysis; n=3, one sample t-test, data are mean ± SEM. (**B**) HeLa cells were treated with control or siRNA against p37 for 48 h, followed by treatment with 400 nM Bafilomycin A1 (BafA1) for 4 h and immunostaining for LC3; n=4, two-tailed Student's t-test, data are mean ± SEM. (**C**) Control and p37 knockout HeLa cells were treated with 400 nM Bafilomycin A1 (BafA1) for 4 h where indicated, followed by western blot analysis; n=3, two-tailed Student's t-test, data are mean ± SEM. (**D**) Protein lysates from human iPSC-derived neurons (iNeurons), SH-SY5Y, HEK293 and HeLa cells were analysed by western blot for the levels of p37 and VCP. (**E**) Quantification of p37 levels related to Fig. 3B. iNeurons treated with lentiviral-delivered p37 shRNA (#81 or #83) for 4 days were treated with 400 nM BafA1 for 6 h; n=4, one sample t-test, data are mean ± SEM. (**F**) Control, p37 knockout or p37 knockout HeLa cells expressing wild-type p37-FLAG were incubated in EBSS starvation medium for 2 h, followed by immunostaining for WIPI2 and FLAG; n=3, two-tailed unpaired Student's t-test, data are mean ± SEM. (**G**) Endogenous immunoprecipitation of VCP from p37-FLAG expressing cells, showing (p37 or FLAG specific antibodies) p37-FLAG binding with VCP. Scale bars, 10 µm. In all cDNA transfection experiments, matched empty vectors were used as controls for overexpression constructs. Similarly, in all knockdown experiments we used non-targetting control siRNAs/shRNAs.

Figure S4

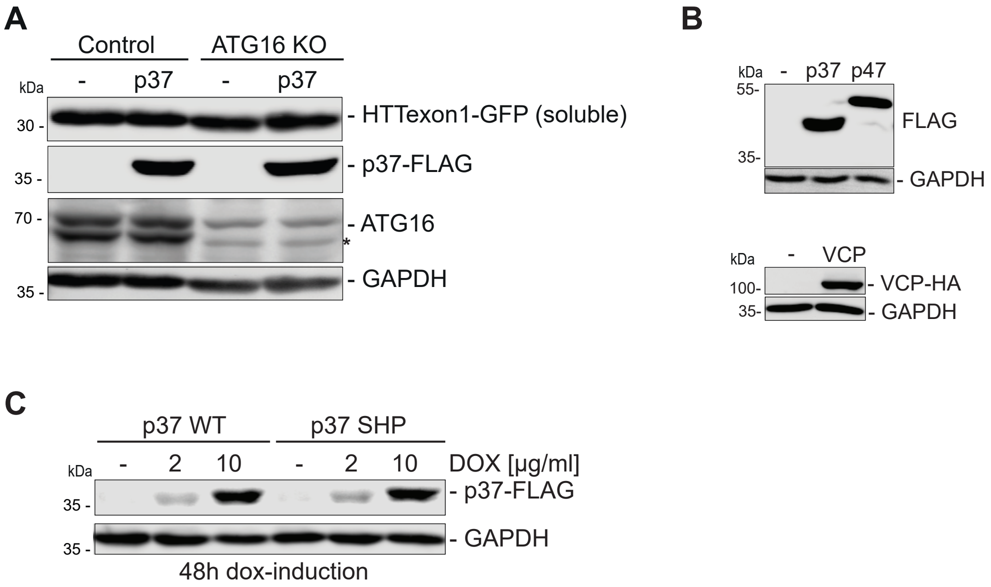
A
Control
ATG16 KO
kDa
-
p37
-
p37
30 -
- HTTexon1-GFP (soluble)
35 -
- p37-FLAG
70 -
- ATG16
*
35 -
- GAPDH
B
kDa
-
p37
p47
55-
35-
FLAG
- GAPDH
kDa
-
VCP
100-
- VCP-HA
35-
- GAPDH
C
p37 WT
p37 SHP
kDa
-
2
10
-
2
10
DOX [µg/ml]
35 -
- p37-FLAG
35 -
- GAPDH
48h dox-induction

Fig. S4. p37 regulates clearance of aggregate-prone proteins.

(**A**) Related to Fig. 4B, showing an equal expression of soluble HTTQ74-EGFP at 24 h in control and ATG16 knockout cells expressing p37-FLAG. (**B**) Related to Fig. 4C, showing expression of p37-FLAG, p47-FLAG and VCP-HA. (**C**) Related to Fig. 4D. A53T-SNCA-EGFP HeLa cells stably expressing wild-type (WT) p37-FLAG or p37 SHP-FLAG mutant under doxycycline-induced promoter were treated with increasing concentrations of doxycycline for 48 h, prior to western blot analysis; *, unspecific band. In all cDNA transfection experiments, matched empty vectors were used as controls for overexpression constructs.

Figure S5

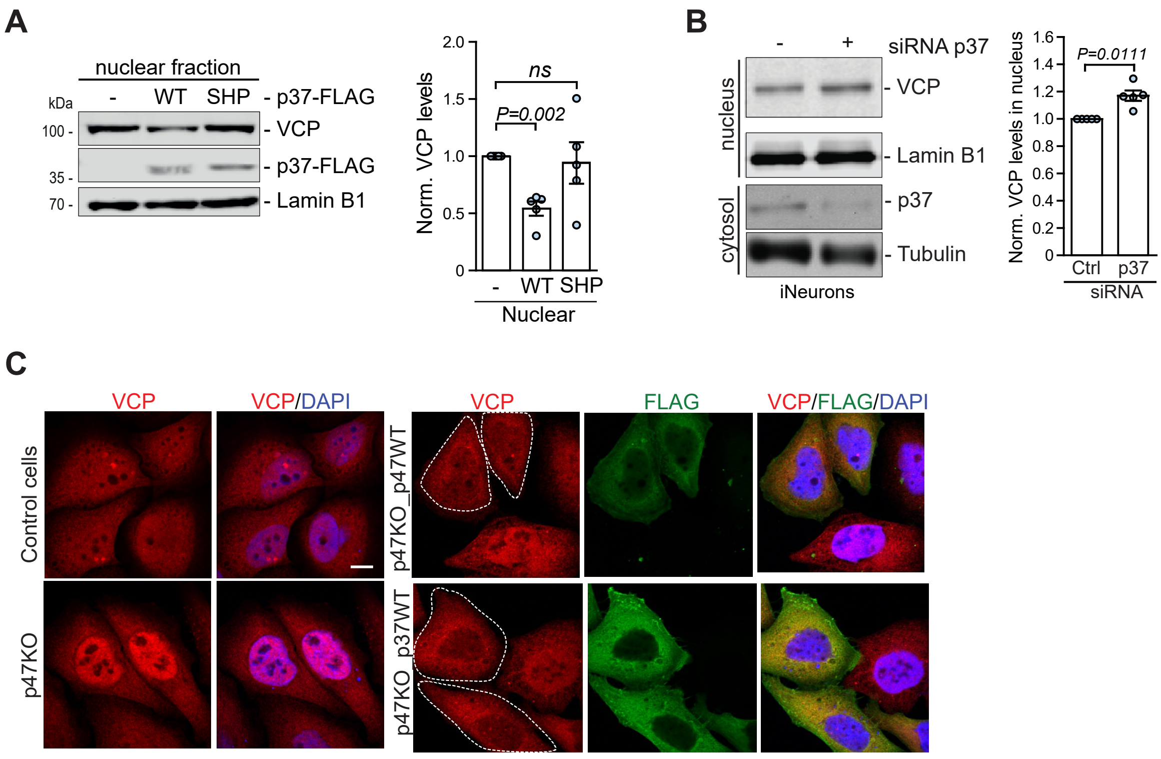

A
nuclear fraction
kDa
- WT SHP - p37-FLAG
100 -
- VCP
35 -
- p37-FLAG
70 -
- Lamin B1
Norm. VCP levels
2.0
1.5
1.0
0.5
0
ns
P=0.002
- WT SHP
Nuclear
B
- + siRNA p37
nucleus
- VCP
- Lamin B1
cytosol
- p37
- Tubulin
iNeurons
Norm. VCP levels in nucleus
1.6
1.4
1.2
1.0
0.8
0.6
0.4
0.2
0
P=0.0111
Ctrl p37
siRNA
C
VCP
VCP/DAPI
VCP
FLAG
VCP/FLAG/DAPI
Control cells
p47KO
p47KO_p47WT
p47KO_p37WT

Fig. S5. p37 regulates VCP nucleocytoplasmic shuttling.

(**A**) Nuclear fraction from HeLa cells expressing wild-type (WT) p37-FLAG or p37 SHP-FLAG mutant was analysed by western blotting for VCP levels, with Lamin B1 as a nuclear marker; n=4, one sample t-test, data are mean ± SEM. (**B**) iNeurons treated with lentiviral-delivered p37 shRNA (#83) for 4 days were fractionated into cytosolic and nuclear fractions, followed by western blot analysis for VCP levels, with Lamin B1 as a nuclear marker; n=5, one sample t-test, data are mean ± SEM. (**C**) Control, p47 knockout and p47 knockout HeLa cells expressing wild-type (WT) p37-FLAG or wild-type((WT) p47-FLAG were immunostained for VCP and FLAG. Scale bars, 10 µm. In all cDNA transfection experiments, matched empty vectors were used as controls for overexpression constructs. Similarly, in all knockdown experiments we used non-targetting control siRNAs.

Figure S6

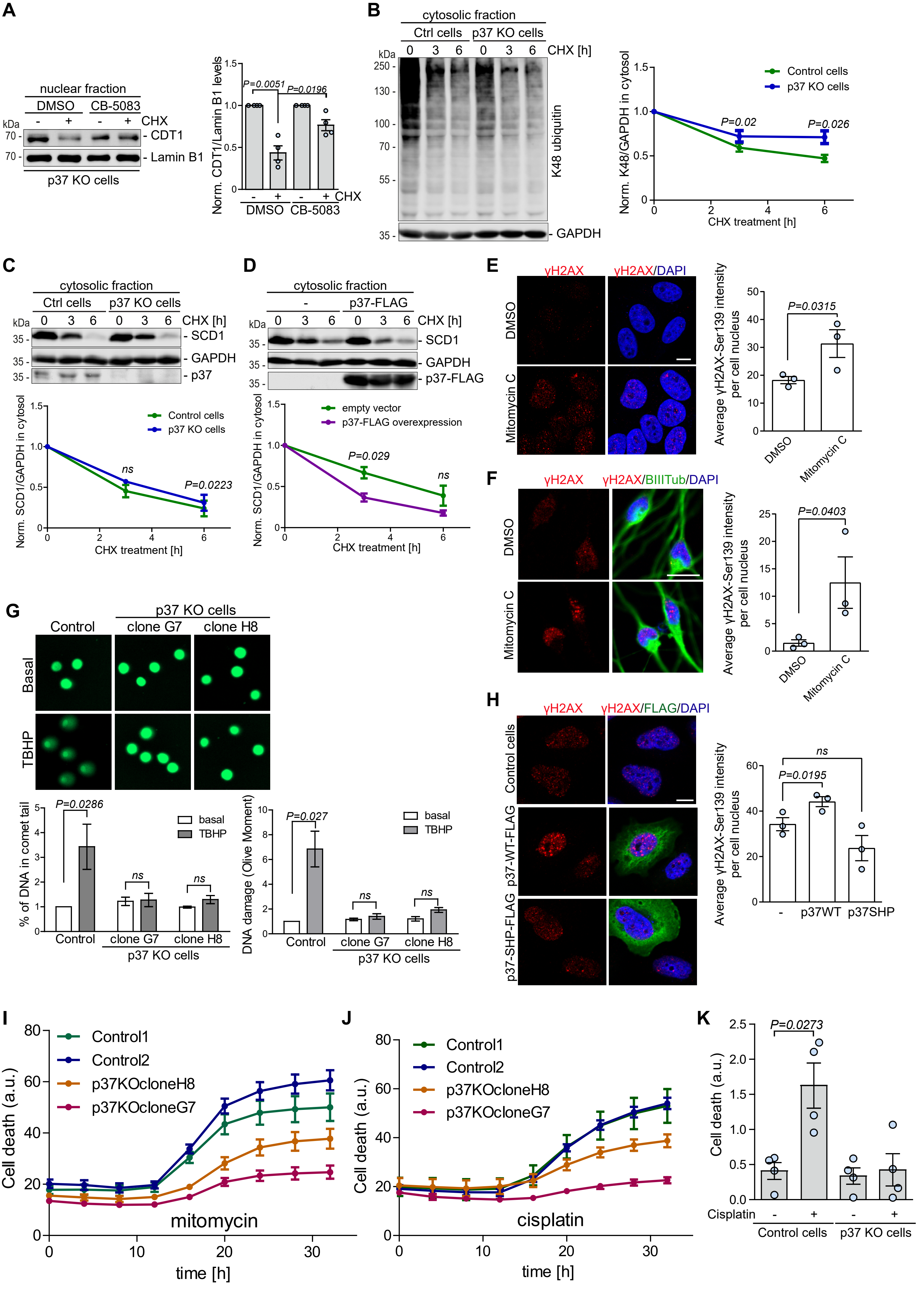
A
nuclear fraction
DMSO
CB-5083
CHX
CDT1
Lamin B1
p37 KO cells
Norm. CDT1/Lamin B1 levels
P=0.0051
P=0.0196
B
cytosolic fraction
Ctrl cells
p37 KO cells
CHX [h]
K48 ubiquitin
GAPDH
Control cells
p37 KO cells
Norm. K48/GAPDH in cytosol
P=0.02
P=0.026
CHX treatment [h]
C
cytosolic fraction
Ctrl cells
p37 KO cells
SCD1
GAPDH
p37
Norm. SCD1/GAPDH in cytosol
ns
P=0.0223
CHX treatment [h]
D
cytosolic fraction
p37-FLAG
SCD1
GAPDH
p37-FLAG
empty vector
p37-FLAG overexpression
P=0.029
ns
CHX treatment [h]
E
γH2AX
γH2AX/DAPI
DMSO
Mitomycin C
Average γH2AX-Ser139 intensity per cell nucleus
P=0.0315
F
γH2AX
γH2AX/BIIITub/DAPI
DMSO
Mitomycin C
P=0.0403
G
Control
p37 KO cells
clone G7
clone H8
Basal
TBHP
% of DNA in comet tail
P=0.0286
ns
basal
TBHP
DNA damage (Olive Moment)
P=0.027
H
γH2AX
γH2AX/FLAG/DAPI
Control cells
p37-WT-FLAG
p37-SHP-FLAG
P=0.0195
ns
p37WT
p37SHP
I
Control1
Control2
p37KOcloneH8
p37KOcloneG7
Cell death (a.u.)
mitomycin
time [h]
J
cisplatin
K
P=0.0273
Cell death (a.u.)
Cisplatin
Control cells
p37 KO cells

fig. S6. p37-mediated VCP shuttling regulates ERAD and DNA damage pathways.

(**A**) p37 knockout HeLa cells were treated with cycloheximide (CHX, 50 μg/ml) for 4 h in the presence or absence of 5 μM CB-5083 VCP inhibitor (with 1 h pretreatment prior CHX addition), followed by nuclear fraction isolation and western blot analysis for CDT1 levels; n=4, one sample t-test. (**B**) Control or p37 knockout HeLa cells were treated with cycloheximide (CHX, 50 μg/ml) for indicated time points, followed by cytosolic fraction isolation and western blot analysis for K48-ubiquitin-conjugated protein levels; n=3, two-tailed Student's *t*-test, data normalised to 0 h time point. (**C**, **D**) Control and p37 knockout (C) or control and p37-FLAG overexpressing HeLa cells (D) were treated with cycloheximide (CHX, 50 μg/ml) for indicated time points, followed by cytosolic fraction isolation and western blot analysis for SCD1 levels, n=3, two-tailed Student's *t*-test, data normalised to 0 h time point. (**E**) Control experiment. HeLa cells were treated with mitomycin C (1 μg/ml) for 2 h. followed by immunostaining for γ-H2AX-Ser319; n=3, one-tailed paired Student's t-test; scale bar, 10 μm. (**F**) Control experiment. iNeurons were treated with mitomycin C (1 μg/ml) for 6 h. followed by immunostaining for γ-H2AX-Ser319; n=3, one-tailed paired Student's t-test; scale bar, 10 μm. (**G**) Alkaline comet assay. Control and p37 knockout cells (clonal line G7 and H8) were treated for 1 h with 100 μM tert-Butyl hydroperoxide (TBHP) and analysed using alkaline comet assay; n=4, two-tailed Student's t-test. (**H**) Control or HeLa cells expressing with wild-type (WT) p37-FLAG or p37 SHP-FLAG mutant were treated with mitomycin C (1 μg/ml) for 2 h, followed by immunostaining for γ-H2AX-Ser319 and FLAG; n=3, two-tailed Student's t-test, scale bar, 10 μm. (**I-K**) Two control lines (Control 1, Control 2) and two p37 knockout lines (p37KOcloneH8, p37KOcloneG7) were treated with mitomycin C (2 μg/ml) (**I**) or 12 μM cisplatin (J,K) for 32 h and cell death was monitored every 4 h, data are plotted as average of CellTox™ Green Dye signal with ± SD of areas analysed per well; statistical analysis in (K), n=4, data are represented as slope values of cell death curve over time, one-way ANOVA (P=0.0029) with post hoc Tukey test. Data are mean ± SEM. In all cDNA transfection experiments, matched empty vectors were used as controls for overexpression constructs.

Figure S7

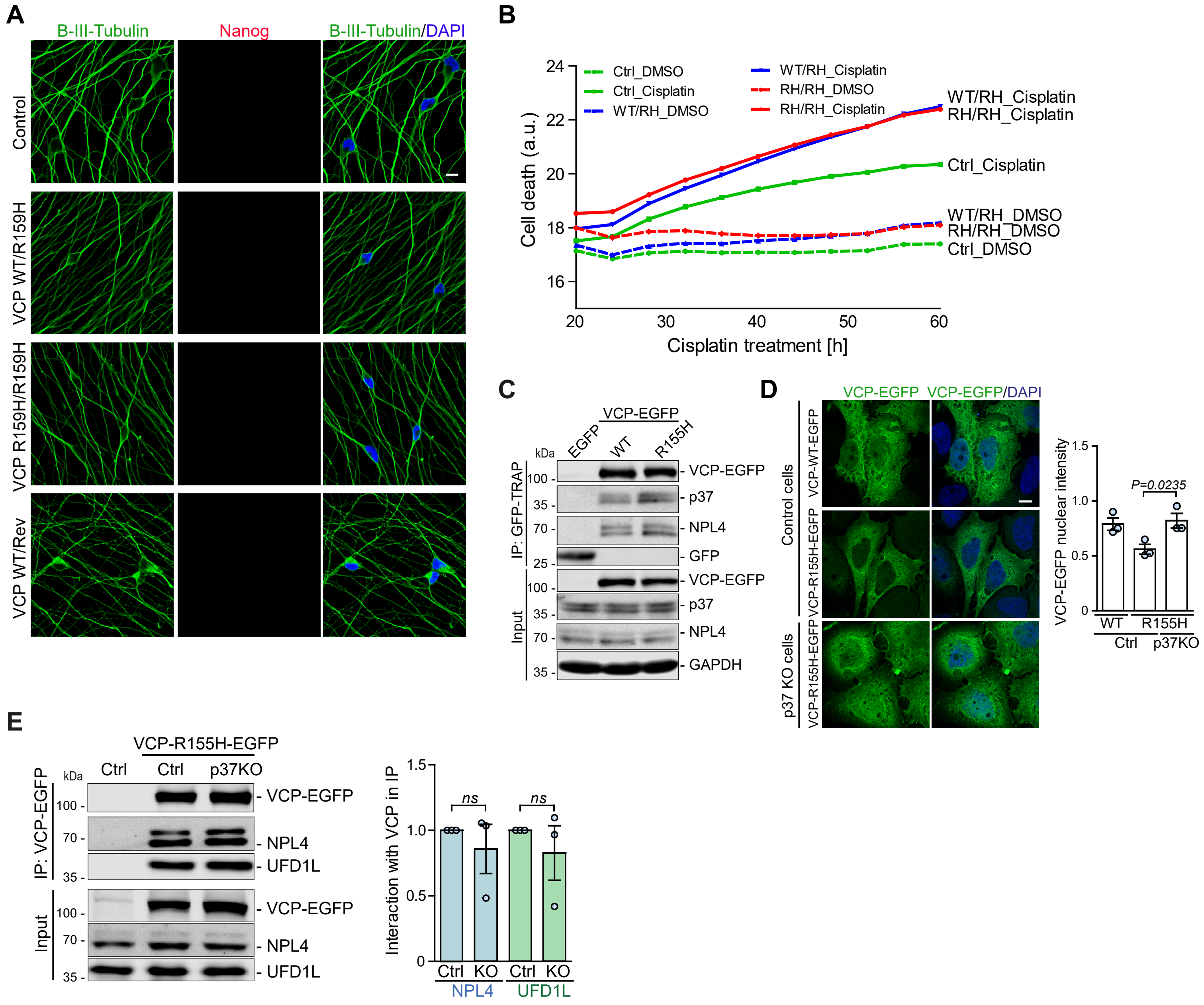
A
B-III-Tubulin
Nanog
B-III-Tubulin/DAPI
Control
VCP WT/R159H
VCP R159H/R159H
VCP WT/Rev
B
Ctrl_DMSO
Ctrl_Cisplatin
WT/RH_DMSO
WT/RH_Cisplatin
RH/RH_DMSO
RH/RH_Cisplatin
Cell death (a.u.)
Cisplatin treatment [h]
WT/RH_Cisplatin
RH/RH_Cisplatin
Ctrl_Cisplatin
WT/RH_DMSO
RH/RH_DMSO
Ctrl_DMSO
C
VCP-EGFP
EGFP
WT
R155H
kDa
IP: GFP-TRAP
Input
VCP-EGFP
p37
NPL4
GFP
GAPDH
D
VCP-EGFP
VCP-EGFP/DAPI
Control cells
VCP-WT-EGFP
VCP-R155H-EGFP
p37 KO cells
VCP-R155H-EGFP
VCP-EGFP nuclear intensity
P=0.0235
WT
R155H
Ctrl
p37KO
E
VCP-R155H-EGFP
Ctrl
Ctrl
p37KO
kDa
IP: VCP-EGFP
Input
VCP-EGFP
NPL4
UFD1L
Interaction with VCP in IP
ns
ns
Ctrl
KO
Ctrl
KO
NPL4
UFD1L

fig. S7. Depletion of p37 restores supressed nuclear localisation of VCP proteinopathy mutant.

(**A**) Control, heterozygous VCP R159H mutant (WT/R159H), homozygous VCP R159H mutant (R159H/R159H) and control revertant (Rev) iNeurons were differentiated for 14 days, followed by immunostaining with neuronal marker β-III-Tubulin and stem cells marker Nanog. No signal from Nanog and positive signal from β-III-Tubulin confirms successful differentiation into iNeurons. (**B**) Representative experiment related to Fig. 7C. Control, heterozygous VCP R159H mutant (WT/R159H), homozygous VCP R159H mutant (R159H/R159H) were treated with DMSO or 12 μM Cisplatin for 60 h and cell death was monitored every 4 h, data are plotted as average of CellTox™ Green Dye signal per well. (**C**) HeLa cells expressing just EGFP, wild-type (WT) VCP-EGFP or VCP-R155H mutant-EGFP were lysed and VCP-EGFP was immunoprecipitated together with its cofactors, followed by western blot analysis. (**D**) Control or p37 knockout cells expressing wild-type (WT) VCP-EGFP or VCP-R155H mutant-EGFP were analysed for the GFP signal in the nuclear compartment; n=3, one-way ANOVA (P=0.0271) with post hoc Tukey test, data are mean ± SEM. (**E**) Control or p37 knockout cells expressing VCP-R155H mutant-EGFP were lysed and VCP-R155H mutant-EGFP was immunoprecipitated together with its cofactors, followed by western blot analysis; n=3, one sample t-test, data are mean ± SEM. Scale bars, 10 μm. In all cDNA transfection experiments, matched empty vectors were used as controls for overexpression constructs.
